# Supplementary figures and images for: Horizontal transmission and recombination maintain forever young bacterial symbiont genomes
Source: PLoS Genet. 2020 Aug 25;16(8):e1008935. doi: 10.1371/journal.pgen.1008935 (PMC7473567; doi:10.1371/journal.pgen.1008935)

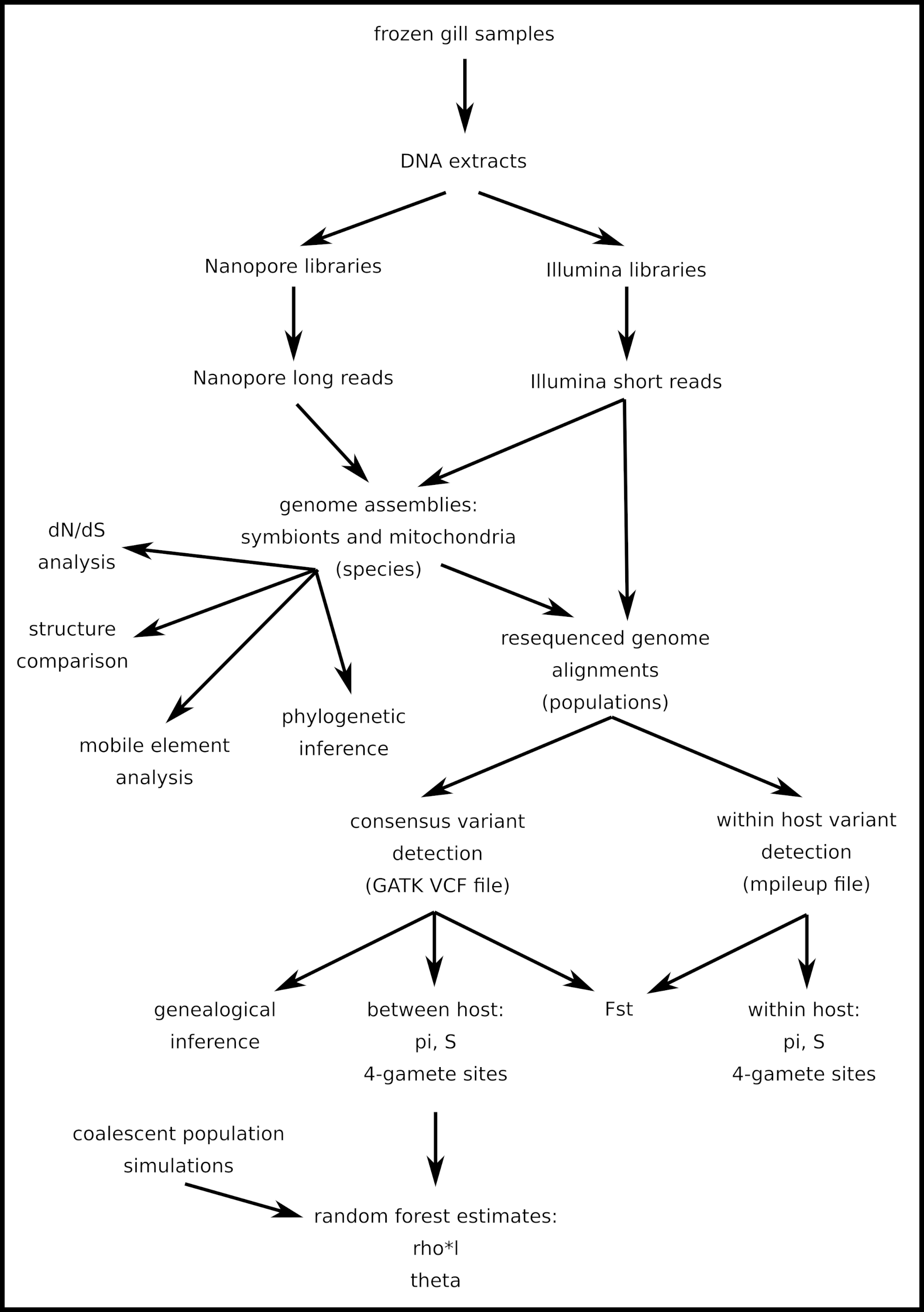

Supplement: S1 Fig — (TIF) [file pgen.1008935.s001.tif]

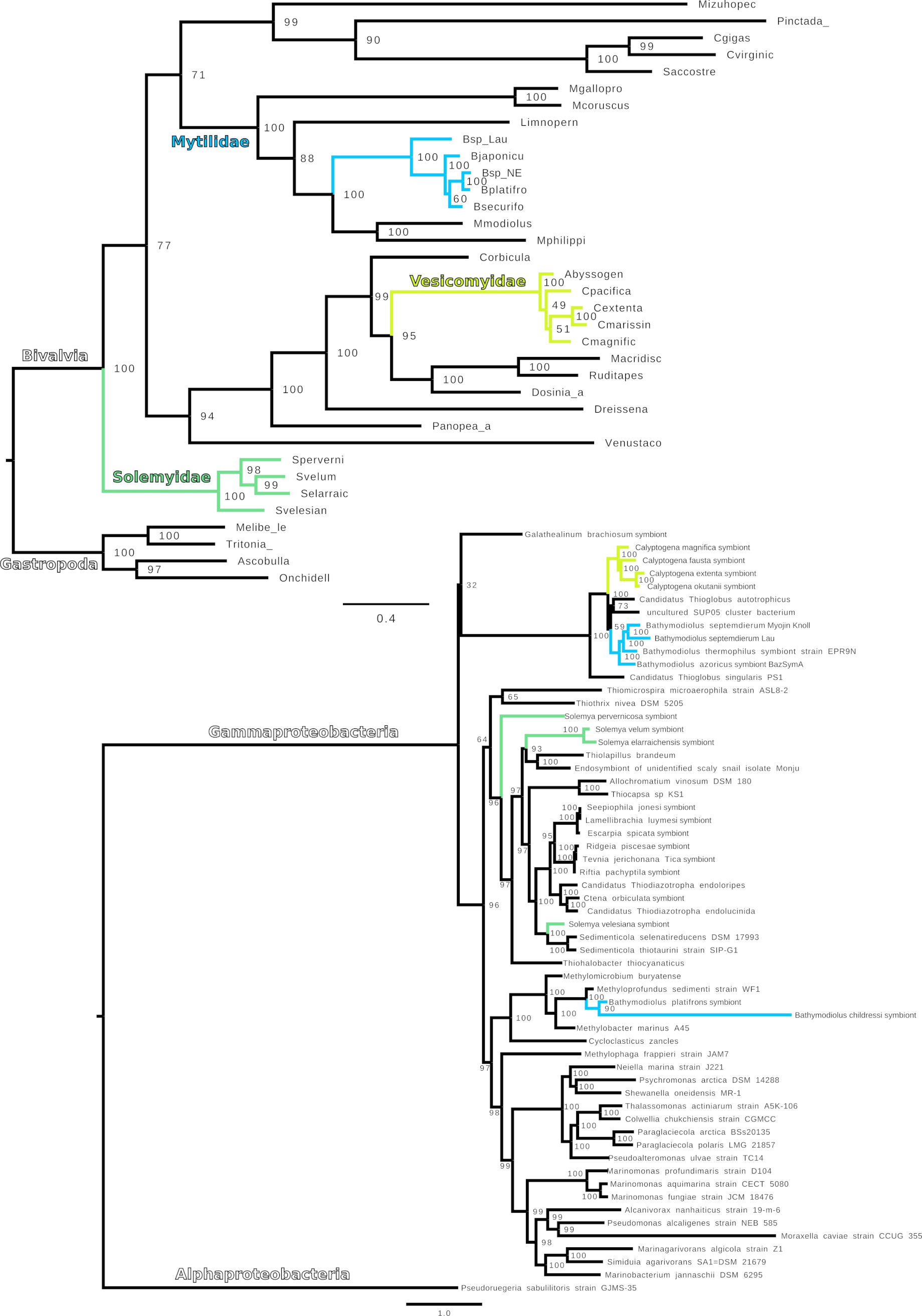

Supplement: S2 Fig — Groups of chemosynthetic associations are colored as in Fig 1: yellow = vesicomyids, green = solemyids, and blue = bathymodiolids. Mitochondrial and symbiont trees are rooted by gastropod and alphaproteobacterial outgroups, respectively. Scale bar = substitutions per site. Bootstrap support values indicated at nodes. (TIF) [file pgen.1008935.s002.tif]

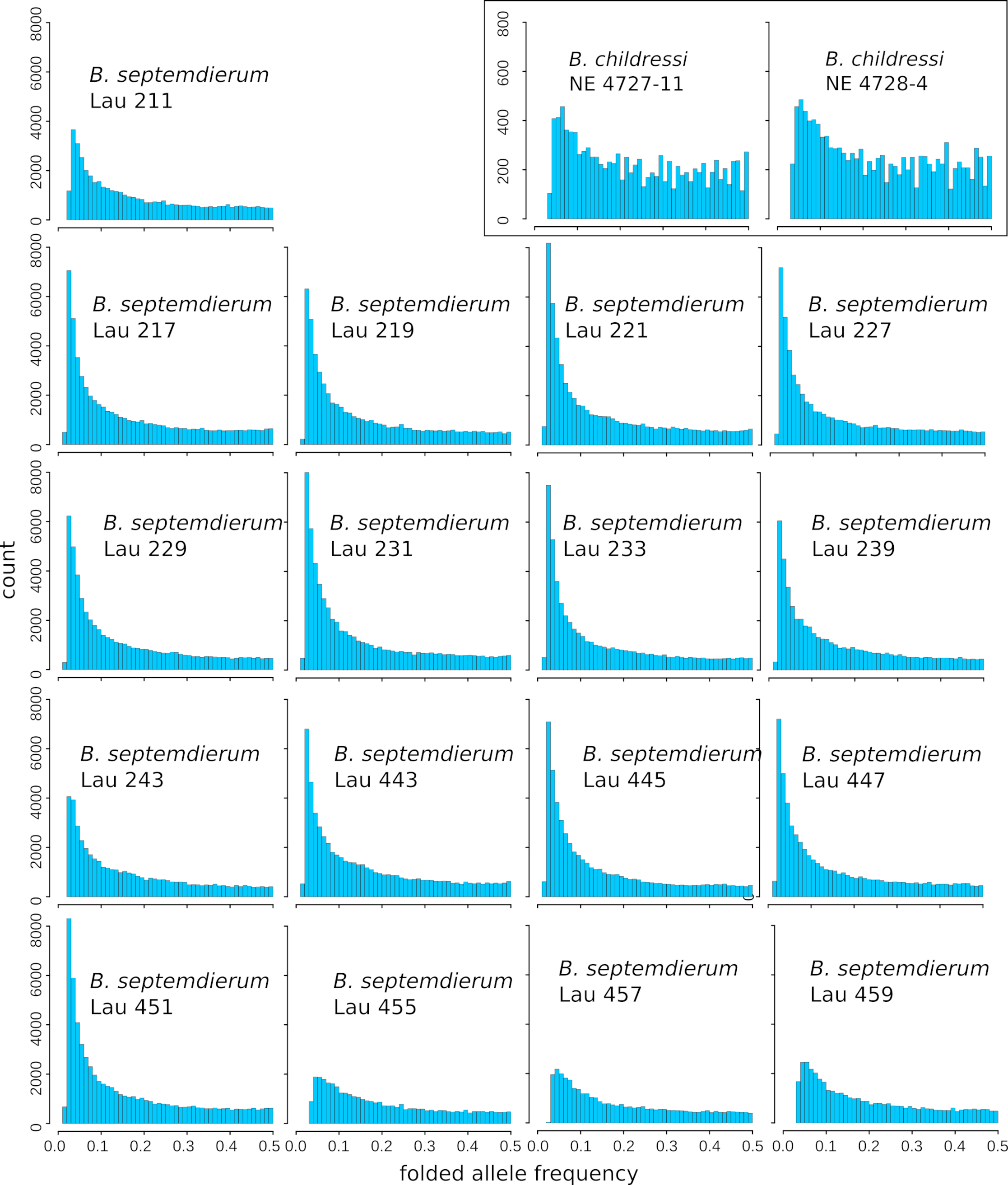

Supplement: S3 Fig — (TIF) [file pgen.1008935.s003.tif]

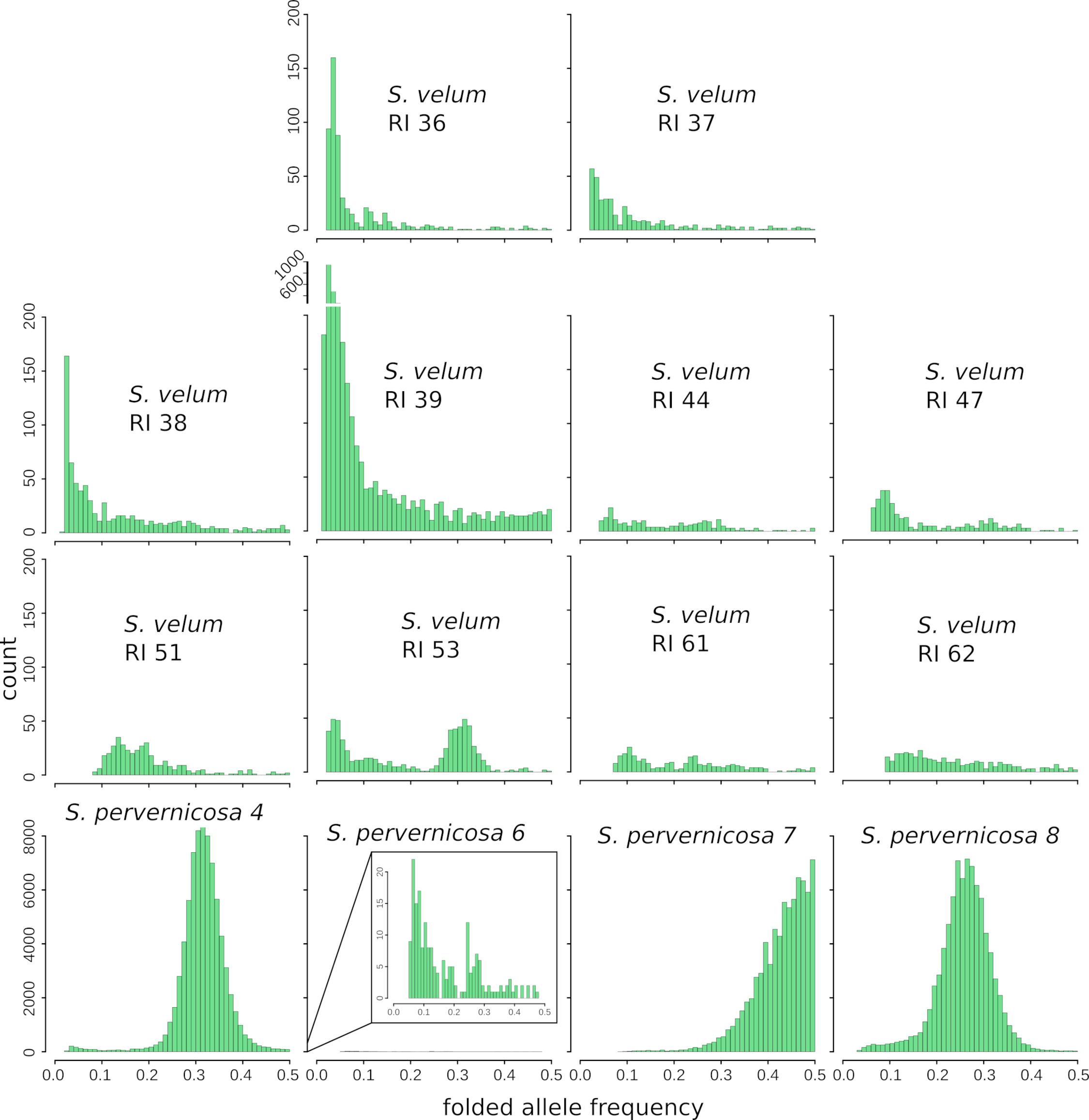

Supplement: S4 Fig — (TIF) [file pgen.1008935.s004.tif]

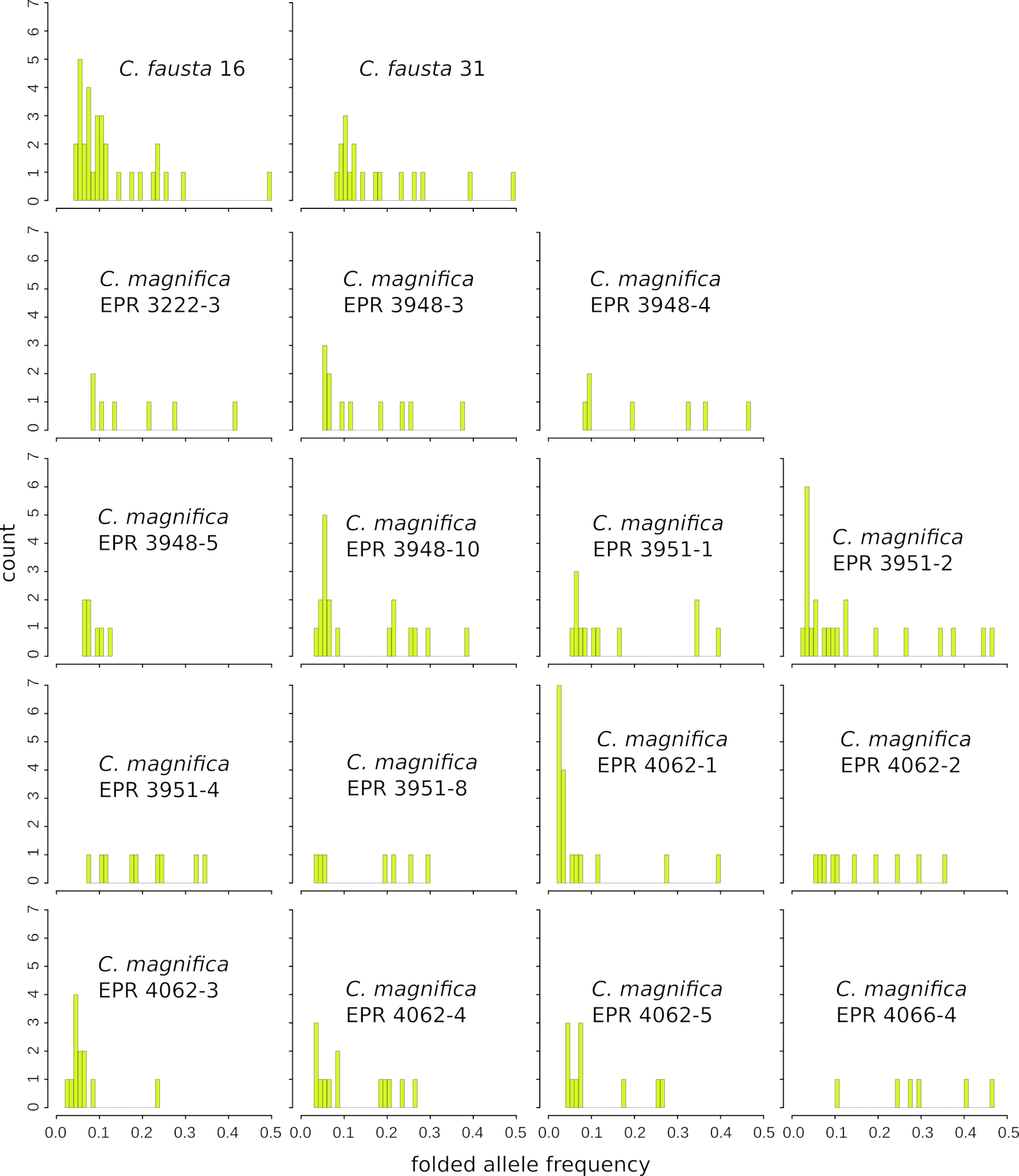

Supplement: S5 Fig — (TIF) [file pgen.1008935.s005.tif]

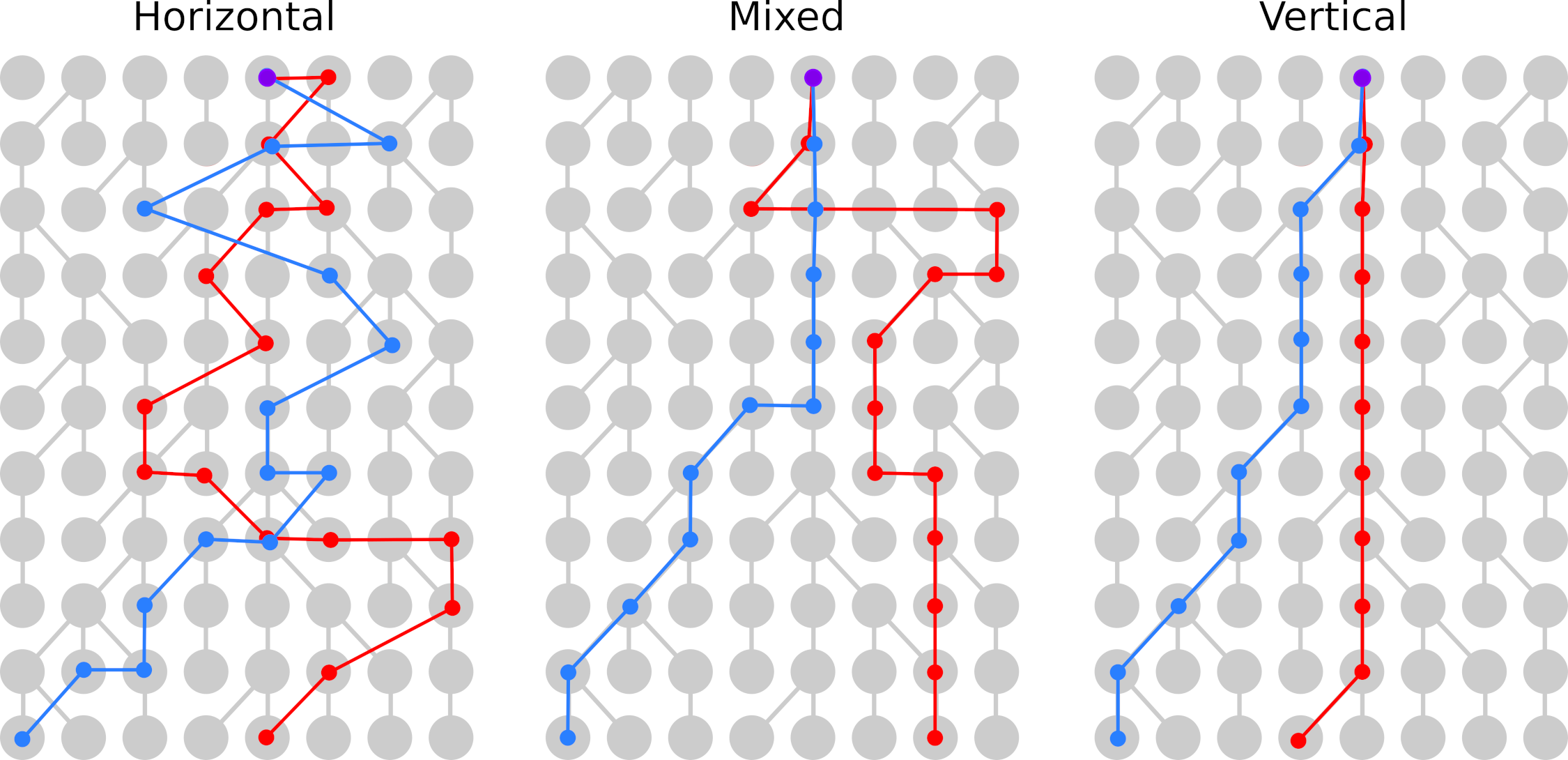

Supplement: S6 Fig — Our generalized coalescent model of endosymbiont inheritance includes symbiont transmission modes ranging from strict horizontal transmission to strict vertical transmission, with mixed modes, exhibiting both horizontal and vertical strategies. The host populations (grey) undergo Wright-Fisher reproduction. Endosymbiont lineages (red and blue) either switch between host lineages or are inherited, depending on the transmission mode, until they coalesce in the same host lineage (purple). (TIF) [file pgen.1008935.s006.tif]

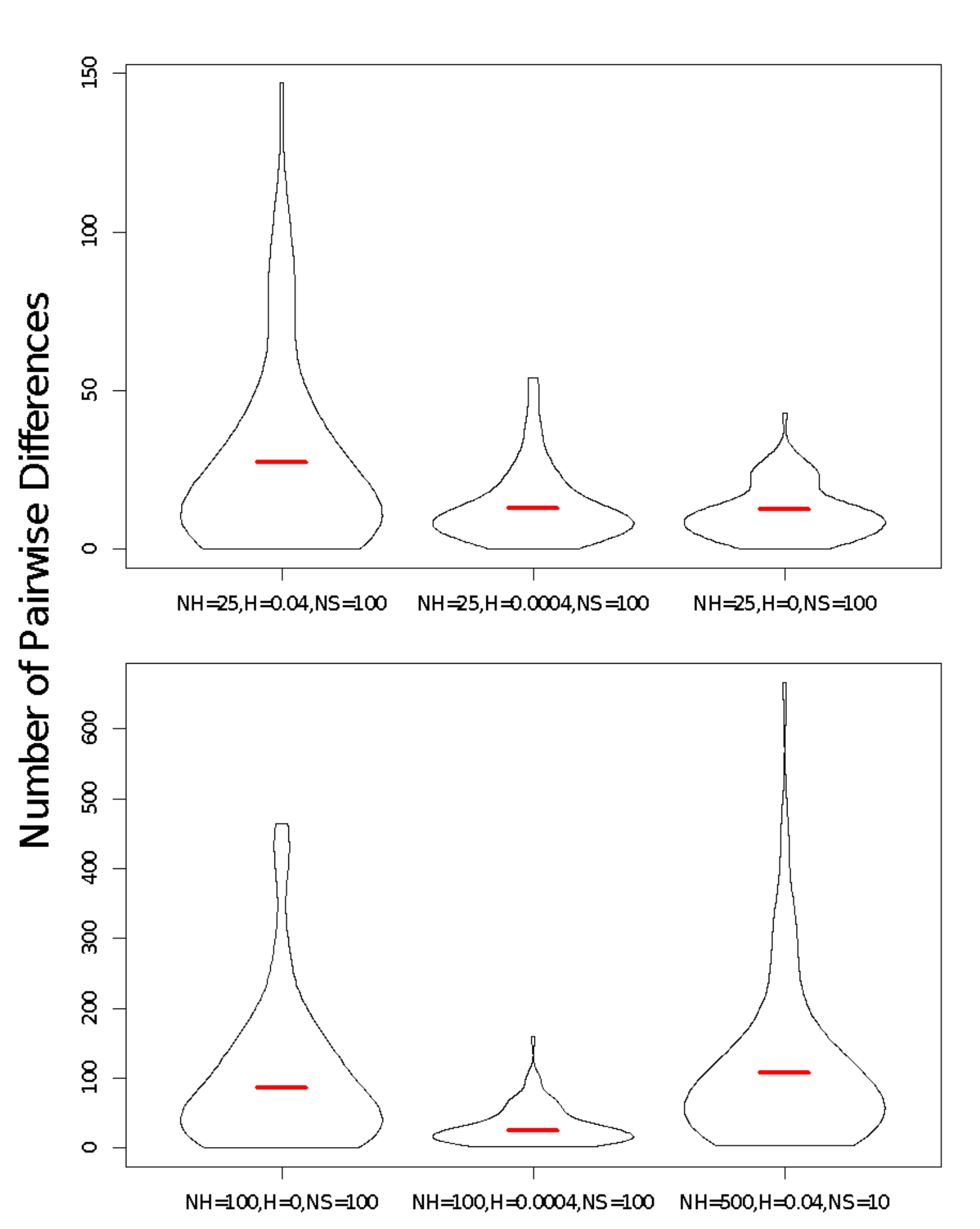

Supplement: S7 Fig — Each distribution is 100 replicates with varying NH, H, and NS. The expectation following Equation 9 above is plotted as a red line and differs by less than 2 segregating sites from the observed mean for all cases investigated here. (TIF) [file pgen.1008935.s007.tif]

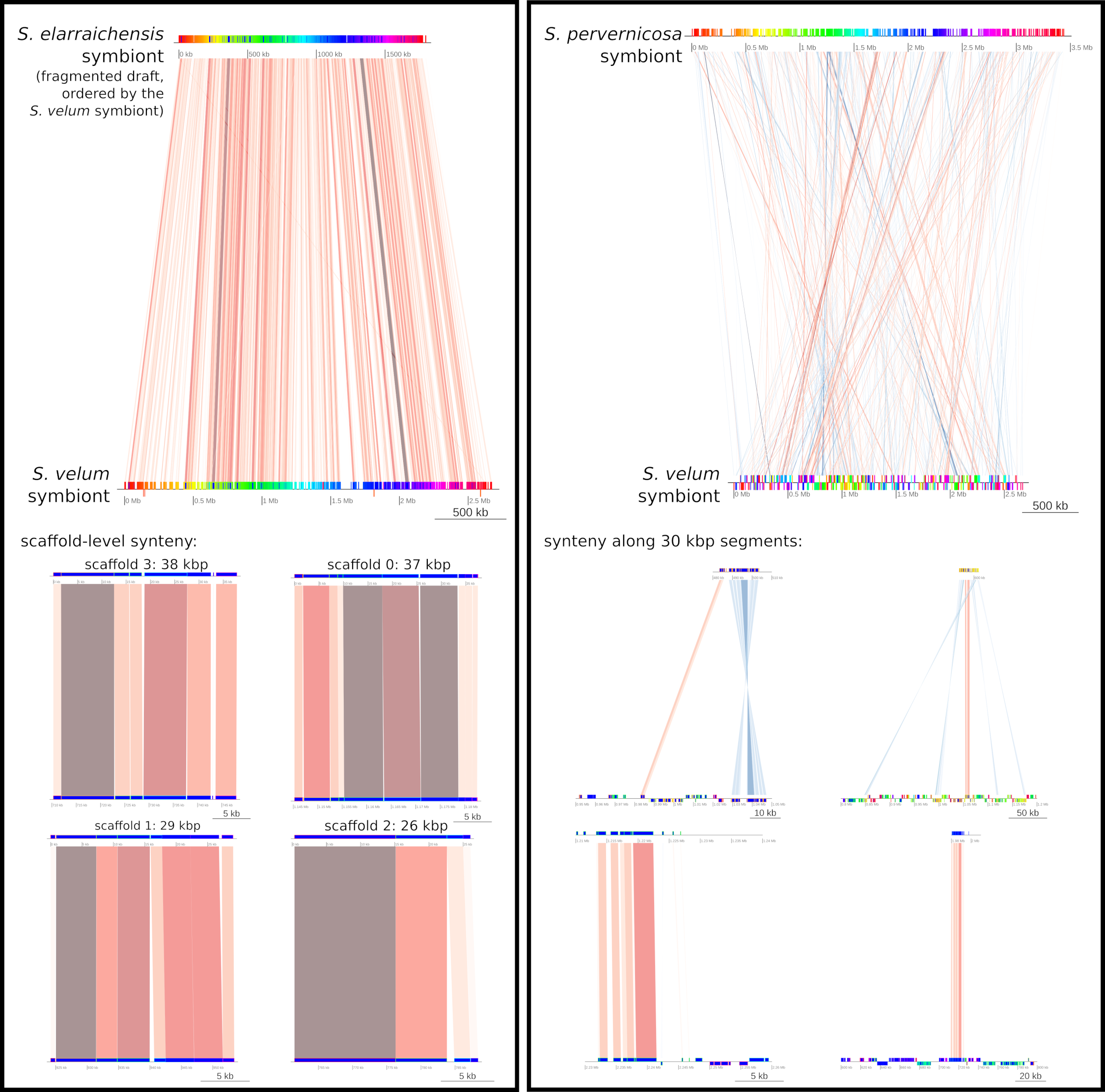

Supplement: S8 Fig — S. elarraichensis symbiont is the closest known relative of the S. velum symbiont, however material is exceedingly hard to obtain for this association, which occurs at a mud volcano at approximately 500–1000 m depth, and only a fragmented draft genome assembly was available. However, even these relatively short range segments reveal complete synteny (left). In comparison, over the same genomic distances, many rearrangements are evident between S. velum and S. pervernicosa (right), with the minority of segments retaining synteny. (TIF) [file pgen.1008935.s008.tif]
