# Supplementary material for: Horizontal transmission and recombination maintain forever young bacterial symbiont genomes
Source: PLoS Genet. 2020 Aug 25;16(8):e1008935. doi: 10.1371/journal.pgen.1008935 (PMC7473567; doi:10.1371/journal.pgen.1008935)
Supplement: S1 Text — (PDF) [file pgen.1008935.s021.pdf]

S1 Text: Forever young bacterial symbiont genomes

Table of Contents

P 1-3	Section 1: Supplemental Methods
P 3-10	Section 2: A Coalescent Model for Endosymbiont Populations with Mixed Transmission Modes
P 10-15	Section 3: Symbiont Species Descriptions
P 15-16	Supporting References

Section 1: Supplemental Methods

Samples and Genomic Data Production, Continued

Verification of species identification
Species identifications for the bivalve samples were confirmed by BLAST. We extracted the full cytochrome oxidase 1 (CO1) gene sequence from each annotated mitochondrial genome to verify the host species. To verify the symbiont species/strain, we extracted the full 16S rRNA sequence from the annotated symbiont genomes. We then blasted both sequences against the non-redundant nucleic acid database from NCBI to find the closest sequence match.

We found that our *B. septemdierum* sequence is 99.87% similar to the other known *B. septemdierum* sequence (100% query coverage). Our *B. childressi* sequence too is nearly identical to the known *B. childressi* sequences, at 99.54-99.93% sequence identity (100% query coverage). Similarly, the symbionts of these two host species were most closely related at the 16S to the symbiont of *B. septemdierum* str. Myojin knoll (99.28% identity and 100% coverage) and *B. childressi* (99.53% identity and 98% coverage).

The *S. velum* samples were sequenced and identified previously [9], and the *S. pervernicosa* in that study came from the same sample set as the *S. pervernicosa* samples sequenced in this paper. Nevertheless, we compared the assembled symbiont 16S sequence against NCBI and found that the previously sequenced and assembled *S. pervernicosa* symbiont was the best hit (100% identity over 100% of query), and the other *S. pervernicosa* or *reidi* (synonym) sequences were >99.5% identical as well.

Although they were collected off of Oregon (JDF Ridge) and morphologically identified as *Calyptogena pacifica*, our *C. fausta* samples were identified as such by their CO1 sequence similarity of 97.32% to *C. fausta* in NCBI, as compared to a similarity of 91.63% to *C. pacifica* (both with 100% query coverage). Interestingly, the assembled symbiont 16S sequence was 100% identical to the *C. fausta* symbiont in NCBI (over 98% of the query) and it was also

99.86% identical to the *C. pacifica* symbiont (over 92% of the query). These two sequences were also very similar to each other (99.86% identical over 94% of the *C. fausta* sequence), indicating these symbionts are very closely related.

Lastly, we blasted the assembled *C. extenta* mitochondrial CO1 sequence to the NCBI database and found that this sample matches the NCBI sequences at 99.29-100% sequence identity (over amplicon fragments covering 33-42% of the query). Comparing the symbiont 16S sequence assembled from this sample to the database resulted in a hit to the C. extenta symbiont at 100% sequence identity (across 92% of the query).

Genotyping error rate estimation

Due to an inadvertent demultiplexing error for the single lane containing *C. fausta* samples, each sample was demultiplexed as a singly, i5, indexed sample. This led to an obvious increase in the rates of index hopping and therefore precluded confident analysis of within individual variation for these samples. Although we expect that consensus genome sequences would be largely unaffected, we decided to confirm this experimentally. We therefore prepared fresh libraries for four of these samples that we sequenced with other libraries and that were demultiplexed using both forward and reverse indexes (second coverage listed in S1 Table). In comparing the four symbiont genome assemblies with those from our initial effectively singly indexed run, we found only a single SNP difference across all four suggesting that our consensus assembly quality is in the range of Q67, or extremely accurate. We therefore used these four libraries for inter-individual variation analyses (S8 Table) and the original ten sequenced individuals for all consensus sequence-based analyses.

Divergence Dating, Continued

Phylogeny inference and divergence date estimation, continued:

To empirically assess what Beast2 divergence estimation parameters to use and how many calibration points to include, we first tested a range of settings run over a MCMC chain length of 100e6 using a HKY model of substitution and a random local molecular clock. We tested the results generated under Yule (birth-only) versus birth-death models of speciation with either a single calibration point at the root of Bivalvia (see Materials and Methods). We also tested multiple calibration points along the host tree for the nodes with fossil data available in the Fossilworks database [106] (i.e., Veneridae, Vesicomyidae, *Calyptogena,* Mytilidae, Ostreidae, Bathymodiolus, Modiolus, Mytilus, Crassostrea, and Solemya). To further assess the impact of node calibration on our results, we also tested uniform versus log-normal prior distributions for the node dates.

While an initial chain length of 100e6 was adequate for ascertaining that the single calibration point strategy was far superior to multiple calibration points in Beast2, the posterior distributions had not stabilized by this point (as visualized in Tracer [116]), so we ran the chains an order of

magnitude longer, which produced well-sampled, converged prior distributions for all settings. We repeated these single calibration (Bivalvia or Gammaproteobacteria) runs for the four pairwise combinations of speciation model (Yule or birth-death) and prior node age distribution (uniform or lognormal) to ensure that the prior distributions converged on the same value when starting from a different seed. Trees were summarized as a maximum clade credibility tree with mean node heights in TreeAnnotator [105], with a 25% burn-in. While all parameter values resulted in similar results, the results from the Yule model with calibration dates modeled with log-normal distribution produced divergence dates most similar to expectations based on fossil evidence (see S6 Table).

We also inferred divergence times using maximum likelihood phylogenies and the RelTime relative rate framework implemented in MEGA [95,96]. This approach produced exceptionally similar values to Beast2, given that it took a fraction of the time to run and uncertainty in the tree was not considered. Given this speed, we were able to test multiple substitution models and models of rate heterogeneity (listed in S6 Table). RelTime's fast inference times also enabled us to estimate divergence dates for symbionts, which were problematic to infer in Beast2 (discussed in the Main Text). We used a single calibration point at the base of the ingroup, Gammaproteobacteria (see Materials and Methods). While some work has been done to infer bacterial divergence dates, few of the bacterial clades in our tree have sufficient evidence and/or genomic taxonomic sampling to warrant inclusion, so we did not test additional bacterial calibration points. In addition to RelTime, we also tried dating mitochondrial and symbiont maximum likelihood phylogenies in PATHd8 [69], which estimates node ages from the mean path lengths between nodes and leaves of these trees. However, PATHd8 did not perform nearly as well as RelTime for both hosts and mitochondria or Beast2 for mitochondria. See S6 Table for divergence date estimates from these different approaches and settings.

Section 2: A Coalescent Model for Endosymbiont Populations with Mixed Transmission Modes

Model Overview

We consider the coalescent process that relates a sample of **n** endosymbiont lineages. Briefly, endosymbiont populations are structured such that all are contained within sub-populations, an "infection" within a single host individual. It is assumed that all symbiont reproduction occurs within a host and that there is a constant rate of horizontal transfer, **H**, between hosts every host generation. In this way, the hosts, numbering $\mathbf{N_H}$ are similar to demes within classical metapopulation models (*e.g.*[117–119]). Hosts are related to one another following a Wright-Fisher model with constant population size and are exchangeable within the model. For simplicity, with the exception of within-host processes, we measure time in host generations. There are therefore three possible states for a pair of symbiont lineages. They may be (1)

occupying different host infections, (2) occupying the same host infection and (3) coalesced (S6 Fig.).

Transition Rates and Host Coalescence

Here, we consider the coalescent process for a sample of two symbiont lineages. Under this model, the probability of a horizontal transmission event that brings a symbiont lineage from one host maternal lineage into another that contains the other symbiont lineage is then

$$p(hori\_trans) = \frac{2H}{N_H} \qquad \text{Equation 1}$$

Here we assume that the H is sufficiently small that H^2 terms can be safely ignored. If we don't make this assumption, this probability would instead be (2H-H^2)/NH. We note that due to the exchangeability of the host lineages, we can ignore all horizontal transmission events that do not bring two symbiont lineages into a single host. In the context of this model, those events are unobservable. However, two lineages may also transition from two distinct hosts to the same in the previous host generation due to coalescence among the host maternal lineages as well.

$$p(host\_coal) = \frac{1}{N_H} \qquad \text{Equation 2}$$

giving a total probability of two lineages transitioning from two separate host infections into a single host during a single host generation.

$$p(same\_host) = \frac{2H+1}{N_H} \qquad \text{Equation 3}$$

This model excludes the possible contribution of male host lineages to coalescence within symbiont populations. If the horizontal transmission probability is very high, this assumption might not be realistic because symbiont lineages could trace their ancestry through males so long as they transmit horizontally within a single host generation. However, if the horizontal transmission rate is relatively low this can be ignored because males will not transmit symbiont populations to their progeny and the majority of sampled symbiont lineages will trace their ancestry almost entirely through maternal host lineages..

Within Host Symbiont Dynamics

We consider that each host begins with $\mathbf{N_S}$ symbiont individuals provisioned by the mother. *I.e.*, this is the size of the transmission bottleneck between host generations. The infection then grows following binary growth for a number of generations $\mathbf{G_S}$. Therefore there are $\mathbf{G_S}$ symbiont

generations per host generation. The probability of coalescence per symbiont generation i in $\{1 \ldots G_S\}$

$$p(coal_i) = \frac{1}{2^{i-1} N_S}$$ Equation 4

for two symbiont lineages within the same host individual that are not coalesced. This ignores the possibility of coalescence after population growth has finished. However, symbiont population sizes within hosts are typically on the order of billions [120–125], and the probability of coalescence is negligible after the first few generations of doubling growth. Therefore, the total probability of symbiont coalescence per host generation for two symbiont lineages that are within the same host, can be approximated as

$$p(coal) = 1 - \prod_{i=1}^{G_s} 1 - \frac{1}{2^{i-1} N_s}$$ Equation 5

We note that for larger values of Ns, this can be approximated as 2/Ns quite well. In the absence of a horizontal transmission event or a coalescent event, both symbiont lineages are transmitted to the host's mother in the previous generation. There is no specific requirement for binary growth within the symbiont population and this process could be modeled more generally using an arbitrary vector of symbiont population sizes if information about symbiont population size dynamics is known (*e.g.* by sampling tissues during development as in [31,33]). However, the smallest symbiont population size will tend to dominate patterns of within host coalescence and therefore estimating the transmission bottleneck inoculate size is likely to be the most pertinent for generating realistic models of symbiont coalescence. For our purposes here, the rate is not particularly consequential.

Alternatively, given that two symbiont lineages are in a single host but have not coalesced, the probability of that one transitions out of the host infection into another host infection is

$$p(diff\_host) = \frac{2H(N_H\text{-}1)}{N_H}$$ Equation 6

Therefore, given that two lineages are in the same host, the probability that they coalesce prior to transmitting out of that host maternal lineage is

$$p(coal \mid same\_host) = \frac{p(coal)}{p(coal) + p(transmit\_out)}$$ Equation 7

Time to Coalescence

In this model, and consistent with most symbiont datasets we are aware of, we consider the case where two symbiont lineages are sampled from different hosts. The number of times that one of the two lineages will enter the same host as the other is geometrically distributed with mean 1/p(coal|same_host), and the expected time until they coalesce, measured in host generations, is the product of the expected number of these events that occur prior to coalescence multiplied by the expected time for each event that brings the two lineages into the same host and they either coalescences or one lineage moves horizontally into another host infection, *i.e.*,

$$E[t_{coal}] = \frac{1}{p(coal \mid same\_host)}\left(\frac{1}{p(same\_host)} + \frac{1}{p(coal) + p(transmit\_out)}\right)$$ Equation 8

Assuming a Poisson distributed per symbiont generation mutation rate μ and an infinite sites model, we obtain the expected number of mutations that separate the two symbiont lineages as

$$E[S] = E[t_{coal}]2\mu G_S$$ Equation 9

Below, we demonstrate that under some conditions these populations can be modeled using Kingman's coalescent, but the above results should hold regardless. Although it is not the primary goal of this work, we also confirmed these predictions using forward-in-time simulations over a range of feasible population conditions (S7 Fig.). This indicates that these equations can correctly predict the nucleotide diversity and time to coalescence for two lineages under arbitrary models of symbiont evolution including those where N_H is of the same order as N_S. Our software to simulate this in forward time is available from https://github.com/shelbirussell/ForeverYoungGenomes_Russell-et-al, but we note that it will generally be much more efficient to use coalescent simulation so long as the population can be modelled appropriately within that framework.

Kingman's coalescent

If the host population size and symbiont inoculate size, N_H and N_S, are of similar magnitude, this model cannot be approximated using Kingman's coalescent and t_{coal} will not be exponentially distributed but rather it will be a mixture of rates. However, for many endosymbiont populations of interest, N_H is likely to be much larger than N_S [121,126–129]. Therefore, the primary rate limiting process for coalescence will be the time until two symbiont lineages are found within the same host maternal lineage, a result closely related to those from metapopulation theory (*e.g.*, [117–119]). Using the framework laid out above, the exact, single host-generation transition matrix, **P,** with probability of coalescence given two lineages are within the same host in the same host generation, **C**, for states (1) two symbiont lineages in different host infections, (2) two symbiont lineages in the same host infection, and (3) coalesced, can be expressed as

$$P = \begin{bmatrix} 1-\frac{(2H+1)}{N_H} & \frac{(2H+1)}{N_H} & 0 \\ \frac{2H(N_H-1)}{N_H} & 1-\frac{2H(N_H-1)}{N_H}-C & C \\ 0 & 0 & 1 \end{bmatrix}$$

Equation 10

We now use the result of Möhle [130,131] to evaluate convergence in Markov processes that operate on two distinct time scales. The above transition matrix, P, contains terms that become increasingly different in the limit as N_H approaches infinity. This matrix can be subdivided into two classes of events. Specifically, F, contains the so-called “fast” events that do not depend on N_H. S contains the “slow” events that depend on the reciprocal of N_H. Finally, an additional term, o(1/N), may contain the terms that trend to zero more quickly than $1/N_H$, however here, this term is equal to zero and omitted below.

$$F = \begin{bmatrix} 1 & 0 & 0 \\ 2H & 1-2H-C & C \\ 0 & 0 & 1 \end{bmatrix}$$

$$S = \begin{bmatrix} -2H-1 & (2H+1) & 0 \\ 0 & 0 & 0 \\ 0 & 0 & 0 \end{bmatrix}$$

Equation 11

Then, if the matrix E = limit(t->infinity)F^t exists, in the continuous approximation of the ancestral process, all of the fast events reach their equilibrium immediately. This can be expressed as

$$E = \begin{bmatrix} 1 & 0 & 0 \\ \frac{2H}{2H+C} & 0 & \frac{C}{2H+C} \\ 0 & 0 & 1 \end{bmatrix}$$

Equation 12

This can be interpreted as follows. Consider a case where two lineages are sampled from the same host (row 2), they will either coalesce or horizontally transfer into two separate host infections essentially immediately with probability equivalent to the relative rates of those possible outcomes. Conversely, if two lineages are already in two separate hosts, they will remain there indefinitely on the time-scale of the “fast” events.

Then, by taking the matrix product, ESE, we obtain the rate matrix G, which in combination with E describes the rescaled ancestral process of the two samples, with time now measured in units of N_H generations. This follows from Möhle's [130,131] theorem, which formally states that this rescaled ancestral process converges to $\lim_{n\to\infty} P^{Nt} = Eexp(tG)$. Below is the rate matrix, G

$$G = \begin{bmatrix} -1-2H+\frac{2H(1+2H)}{C+2H} & 0 & \frac{C(1+2H)}{C+2H} \\ \frac{4H^2(1+2H)}{(C+2H)^2}+\frac{2(-1-2H)H}{C+2H} & 0 & \frac{2CH(1+2H)}{(C+2H)^2} \\ 0 & 0 & 0 \end{bmatrix} \quad \text{Equation 13}$$

This result implies that when two lineages are sampled from different host infections, the rate of coalescence could be further rescaled by (C+2H)/(C(1+2H)), and for most of the plausible parameter space, the total symbiont effective population size will exceed N_H. For example, if C = 0.02, H=0.01, and N_H = 10,000, the symbiont effective population size would be 19,608. This indicates that effective population sizes may sometimes be substantially larger than N_H and is consistent with metapopulation coalescent theory with moderate barriers to migration among small demes [117,118].

Extension to Larger Samples

For the Kingman coalescent to be a valid approximation for evolution of endosymbiont lineages, coalescent events among lineages must be primarily pairwise rather than multiple mergers among several lineages [132–134]. Given the complexity of this model, it is not feasible to evaluate a three or four lineage exact backwards in time transition matrix as we did for two lineages above.

Instead, we make use of the following heuristic observation. In order for symbiont lineages to experience a multiple coalescent event there must be three or more symbiont lineages within a single host at one time. Because we assume that all symbiont lineages are sampled from distinct hosts. Conditional on two lineages occupying the same infection, they will either coalesce or one migrate out with probability p(coal or leave) = C+2H. Conversely, another lineage will enter the same infection with probability p(enter) = (1+H)/N_H. In the limit as N_H trends towards infinity, this value rapidly approaches zero. Because coalescence or departure from the same infection occurs on a much faster time-scale than migration events, all coalescent events must be pairwise in the limiting case, where higher order coalescent events would occur at a rate on the order of 1/N_H^2.

Modeling Recombination

It is increasingly widely appreciated that recombination can have a profound influence on bacterial populations [47,48,135,136]. Throughout, we model recombination as equivalent to occasionally overlapping gene conversion events in eukaryotic species. Moving along a chromosome from left to right, when a gene conversion event occurs on a genealogy, it creates an additional lineage within the converted segment to the right and to the left of the recombination breakpoints (as described in [113]). If no additional gene conversion events occur within the gene conversion tract, then the sampled genome returns to the previous marginal genealogy when the tract ends. Initially, these two symbiont lineages will be present within the same infection and if they coalesce before either lineage transmits into another host maternal lineage, this event will not be observed. Therefore, the observable recombination rate is decreased relative to the per generation recombination rate. Thus, the probability that the recombination event is unobserved due to coalescence within the same infection is $p(\text{unobserved}) = C/(C+2H)$.

There is also a chance that two lineages coalesce prior to coalescing with any other lineages due to subsequent back-migration into the same host maternal lineage, and subsequent coalescence. This would also cause a recombination event to be unobserved and is already an integral part of most coalescent simulation algorithms (*e.g.*, [137,138]), which model the probability of back coalescence to the marginal genealogy to the left. For our purposes it is therefore sufficient to multiply the per-generation recombination rate by a constant factor of $2H/(C+2H)$ to obtain a corrected effective recombination rate estimate in the Kingman coalescent. Importantly, when horizontal transmission is rare relative to within-host coalescence, the effective recombination rate will tend to be quite small for symbiont populations.

Possible Extensions Using Scattering Phase Models

One key implication of this model is that when multiple symbiont lineages are sampled from within a single host, they may sometimes coalesce quickly before either symbiont lineage horizontally transfers into another host maternal lineage. This could feasibly be extended using a separation of time-scales approach as in the “scattering” and “collecting” phases of metapopulation coalescent theory [117–119]. However, we do not believe this addition is necessary for the interpretation of most existing endosymbiont datasets. In general, two lineages that coalesce very quickly will have few polymorphisms that distinguish them. Given current sequencing practices it may be challenging to infer the presence of multiple lineages unless one is a very recent migrant—obviating this concern—and suggesting that these considerations will not significantly affect interpretations of empirical endosymbiont genomic diversity datasets. Nonetheless, as microbial sampling practices continue to progress, with *e.g.* single-cell sequencing approaches becoming more common, modeling these additional considerations may be important for future applications of this framework that do sample multiple lineages from within the same host. However in the analyses presented here, unless otherwise stated, we will sample on a single symbiont lineage from each host individual.

Summary and Assumptions

We have derived a coalescent model for endosymbiont lineages that are partially structured by host populations and experience both horizontal and vertical transmission. We stress that, as with any theoretical framework, there are many assumptions underlying this result and that these assumptions should be critically evaluated when applying these to additional populations and datasets. In particular, this model explicitly assumes that the host population is at a demographic equilibrium and neutrally evolving. Furthermore, we assume that the rate of horizontal transmission is relatively small and that the host population size is much larger than the within-host symbiont effective population size. Although extensions are possible to accommodate these and other added complexities, they are beyond the scope of our treatment here where we focus primarily on understanding the evolution of the endosymbiont populations during mixed transmission modes. However, importantly for this work, this model justifies our use of standard Kingman coalescent simulation software, as it should approximate the evolution of endosymbiont populations during mixed transmission modes and provides a theoretical justification for the mechanistic relationships between horizontal transmission and observable recombination events that have the potential to create genetically diverse symbiont chromosomes.

Section 3: Symbiont Species Descriptions

Name: *Candidatus* Thiodubiliella gen. nov.
Thī.ō.dŭb.ĭlĭ.ĕllă N.L. adj. *thio* sulfur*;* N.L. n. *dubiliella* named for Prof. Nicole Dubilier who has worked on the bathymodiolin deep-sea symbioses throughout her career and contributed significant knowledge to the field about their biology; N.L. fem. n. *Thiodubiliella.*
Properties: Bacterial symbionts in the SUP05 clade of Gammaproteobacteria that associate with deep-sea mytilid mussels at hydrothermal vents. Short, rod-shaped bacteria (~0.6 µm long) live intracellularly within specialized host gill cells [137]. Their genomes encode genes for sulfide and hydrogen oxidation (see *Bathymodiolus septemdierum* symbiont from Lau genome annotation under NCBI BioProject number PRJNA562081 and [42]). Genus diagnosed molecularly by phylogenetic divergence (Figure 5A) and high sequence identity (>95%) at rRNA, core metabolic, and housekeeping genes. Ideally, average nucleotide identity across the genome is >73% for members of the genus (as in [138]). However, if high sequence identity at conserved elements is observed, this constraint can be relaxed (because symbiotic genomes often have increased structural dynamics due to bouts of inefficient selection). Genome structure is indeed dynamic in this clade, leading to much higher divergence at the genome level than the corresponding 16S rRNA level (*e.g.*, 33.1% and 98.6% identity, respectively, between *Ca.* Thiodubiliella symbionts from *Bathymodiolus septemdierum* (Lau) and the unnamed symbionts from *Bathymodiolus thermophilus* (NCBI accession GCF_003711265.1)). The genus exhibits approximately 35% and 95% sequence similarity at the genome an 16S

levels, respectively, to *Candidatus* Thioglobus autotrophicus (unclassified Gammaproteobacteria; NCBI accession GCF_001293165.1) in the closest related genus (see Figure 5A).
Typification: Class: Gammaproteobacteria; Order: unclassified.
Type species: *Candidatus* Thiodubiliella endoseptemdiera sp. nov.

Name: *Candidatus* Thiodubiliella endoseptemdiera sp. nov.
ĕn.dō.sĕpt.ĕm.dĭēr.ă Gr. fem. adj. *endo* within; N.L. n. *septemdiera* (host) *Bathymodiolus septemdierum*; N.L. fem. n. *endoseptemdiera*.
Properties: Intracellular thioautotrophic bacterial gill symbiont of bathymodiolin mussels. Species affiliation defined molecularly by divergence (Figure 5), by ≥95% average nucleotide identity genome-wide, and ≥98.7% sequence identity at rRNA, core metabolic, and housekeeping genes in the genome (*Bathymodiolus septemdierum* symbiont genome annotation under NCBI BioProject number PRJNA562081).
Type host: *Bathymodiolus septemdierum* (Bivalvia: Pteriomorphia: Mytilidae).
Other hosts: None.
Type locality: Tu'i Malila, Lau Basin, S21° 59.3455′ W176° 34.0926′.
Other localities: None.
Specimens deposited: None, as this bacterium is currently unculturable and is only known molecularly and microscopically. This is a tentative name proposed with the designation "*Candidatus*".

Name: *Candidatus* Methanofisheria gen. nov.
Mĕth.ănō.fĭsh.ĕr.ă N.L. adj. *Methano* methane; N.L. n. *fishera* named for Prof. Charles Fisher who has worked on the bathymodiolin deep-sea symbioses throughout his career and contributed significant knowledge to the field about their biology; N.L. n. fem. *Methanofisheria*.
Properties: Bacterial symbionts in a clade of methane-oxidizing Gammaproteobacteria that associate with deep-sea mytilid mussels at hydrothermal vents and cold seeps. Coccoid-shaped bacteria (1.4-2 μm in diameter) containing stacked membranes live intracellularly within specialized host gill cells [139]. Their genomes encode pathways for using methane as both a carbon and an energy source (see *Bathymodiolus childressi* symbiont genome annotation under NCBI BioProject number PRJNA562081), and hosts have been shown to uptake methane [140]. Genus diagnosed molecularly by phylogenetic divergence (Figure 5A) and high sequence identity (>95%) at rRNA, core metabolic, and housekeeping genes. Ideally, average nucleotide identity across the genome is >73% for members of the genus (as in [138]). However, if high sequence identity at conserved elements is observed, this constraint can be relaxed (because symbiotic genomes often have increased structural dynamics due to bouts of inefficient selection). Genome structure is indeed dynamic in this clade, leading to much higher divergence at the genome level than the corresponding 16S rRNA level (*e.g.*, 58.3% and 97.8% identity, respectively, between *Ca.* Methanofisheria symbionts from *Bathymodiolus childressi* and the unnamed symbionts from *Bathymodiolus platifrons* (NCBI accession GCF_002189065.1)). The genus exhibits approximately 50.2% and 94.0% sequence similarity at the genome and 16S levels, respectively, to *Candidatus* Methylobacter oryzae

(Gammaproteobacteria: Methylococcales: Methylococcaceae; NCBI accession GCF_003994235.2) in the closest related genus (see Figure 5A).
Typification: Class: Gammaproteobacteria; Order: unclassified.
Type species: *Candidatus* Methanofishera endochildressiae sp. nov.

Name: *Candidatus* Methanofishera endochildressiae sp. nov.
ĕn.dō.chíld.rĕs.sĭ.āĕ Gr. adj. *endo* within; N.L. n. *childressiae* (host) *Bathymodiolus childressi*.
Properties: Intracellular methanotrophic bacterial gill symbiont of bathymodiolin mussels. Species affiliation defined molecularly by divergence (Figure 5), by ≥95% average nucleotide identity genome-wide, and ≥98.7% sequence identity at rRNA, core metabolic, and housekeeping genes in the genome (*Bathymodiolus childressi* symbiont genome annotation under NCBI BioProject number PRJNA562081).
Type host: *Bathymodiolus childressi* (Bivalvia: Pteriomorphia: Mytilidae)
Other hosts: None.
Type locality: Veatch Canyon, 39.8061°N 69.5922°W
Other localities: None.
Specimens deposited: None, as this bacterium is currently unculturable and is only known molecularly and microscopically. This is a tentative name proposed with the designation "*Candidatus*".

Name: *Candidatus* Cavanaughia gen. nov.
Cāv.ān.ăŭgh.ĭ.ă N.L. n. fem. named for Prof. Colleen Cavanaugh, who was one of the first to discover chemosynthetic symbiosis and worked on the *S. velum* symbiosis throughout her career and contributed significant knowledge to the field about their biology.
Properties: Gammaproteobacterial symbionts that associate with burrowing solemyid bivalves that inhabit continental shelf sediments. Rod-shaped bacteria (2-10 μm long) live intracellularly within specialized host gill cells [141]. Their genomes encode genes for sulfide oxidation (see [13]), and these associations have been shown to oxidize sulfide and fix carbon dioxide [141]. Genus diagnosed molecularly by phylogenetic divergence (Figure 5A) and high sequence identity (>95%) at rRNA, core metabolic, and housekeeping genes. Ideally, average nucleotide identity across the genome is >73% for members of the genus (as in [138]). However, if high sequence identity at conserved elements is observed, this constraint can be relaxed (because symbiotic genomes often have increased structural dynamics due to bouts of inefficient selection). Genome structure is indeed dynamic in this clade, leading to much higher divergence at the genome level than the corresponding 16S rRNA level (*e.g.*, 51.6% and 99.7% identity, respectively, between *Ca.* Cavanaughia symbionts from *Solemya velum* and *Solemya elarraichensis* (NCBI accession GCF_002021095.1)). The genus exhibits approximately 39.9% and 93.1% sequence similarity at the genome an 16S levels, respectively, to *Thiolapillus brandeum* (unclassified Gammaproteobacteria; NCBI accession GCF_000828615.1) in the closest related genus (see Figure 5A).
Typification: Class: Gammaproteobacteria; Order: unclassified.
Type species: *Candidatus* Cavanaughia endovela sp. nov.

Name: *Candidatus* Cavanaughia endovela sp. nov.
ĕn.dō.vē.lă Gr. adj. *endo* within; N.L. *vela* (host) *Solemya velum;* N.L. n. fem. *endovela*.
Properties: Intracellular thioautotrophic bacterial gill symbiont of *Solemya velum* bivalves. Species affiliation defined molecularly by divergence (Figure 5), by ≥95% average nucleotide identity genome-wide, and ≥98.7% sequence identity at rRNA, core metabolic, and housekeeping genes in the genome (NCBI accession GCF_000787395.1; [13]).
Type host: *Solemya velum* (Bivalvia: Protobranchia: Solemyidae)
Other hosts: None.
Type locality: Naushon Island, Woods Hole, MA, USA
Other localities: intertidal-subtidal sediments along the eastern North American coast off: Kingston Bay, Duxbury, MA (42°0'14.15"N, 70°40'43.79"W); Point Judith, Narragansett, RI (41°22'55.91"N, 41°22'55.91"N); Shark River Island, Neptune Township, NJ (40°11'9.60"N, 74° 1'48.00"W); Sinepuxent Bay, MD (38°14'58.56"N, 75°9'8.06"W); Pivers Island, Beaufort, NC (34°42'59.23"N, 76°40'26.33"W).
Specimens deposited: None, as this bacterium is currently unculturable and is only known molecularly and microscopically. This is a tentative name proposed with the designation "*Candidatus*".

Name: *Candidatus* Reidiella gen. nov.
Rēĭd.ĭ.ĕl.lă N.L. n. fem. named for Prof. Robert Reid who first reported on these mouthless and gutless protobranch bivalves (from the synonymized *S. reidi*).
Properties: Gammaproteobacterial symbionts that associate burrowing solemyid bivalves that inhabit continental shelf sediments. Rod-shaped bacteria (2-5 µm long) live intracellularly within specialized host gill cells [30]. Their genomes encode genes for sulfide oxidation (see *Solemya pervernicosa* symbiont genome annotation under NCBI BioProject number PRJNA562081), and these associations have been shown to oxidize sulfide and fix carbon dioxide [142]. Genus diagnosed molecularly by phylogenetic divergence (Figure 5A) and high sequence identity (>95%) at rRNA, core metabolic, and housekeeping genes. Ideally, average nucleotide identity across the genome is >73% for members of the genus (as in [138]). However, if high sequence identity at conserved elements is observed, this constraint can be relaxed (because symbiotic genomes often have increased structural dynamics due to bouts of inefficient selection). Genome structure is indeed dynamic in this clade (Figure 4), however, close relatives of the *Ca.* Reidella symbiont from *S. pervernicosa* have not been sampled. Compared to one of the most closely related species, *Candidatus* Tenderia electrophaga (NCBI accession GCA_001447805.1), *Ca.* Reidella endopervernicosa is 30.4% and 93.50% identical at the whole genome and 16S levels, respectively.
Typification: Class: Gammaproteobacteria; Order: unclassified.
Type species: *Candidatus* Reidiella endopervernicosa sp. nov.

Name: *Candidatus* Reidiella endopervernicosa sp. nov.
ĕn.dō.per.ver.ni.cō.sa Gr. adj. *endo* within; N.L. n. *pervernicosa* (host) *Solemya pervernicosa*; N.L. n. fem. *endopervernicosa.*

Properties: Intracellular thioautotrophic bacterial gill symbiont of *Solemya pervernicosa* bivalves. Species affiliation defined molecularly by divergence (Figure 5), by ≥95% average nucleotide identity genome-wide, and ≥98.7% sequence identity at rRNA, core metabolic, and housekeeping genes in the genome (*Solemya pervernicosa* symbiont genome annotation under NCBI BioProject number PRJNA562081).
Type host: *Solemya pervernicosa* (Bivalvia: Protobranchia: Solemyidae)
Other hosts: syn. *Solemya reidi.*
Type locality: sewage outfall, off Santa Monica, CA, USA
Other localities off Cape Nomamisaki, Kagoshima, Japan (NCBI accession AB499617).
Specimens deposited: None, as this bacterium is currently unculturable and is only known molecularly and microscopically. This is a tentative name proposed with the designation "*Candidatus*".

Name: *Candidatus* Ruthia endofausta sp. nov.
ĕn.dō.făŭ.stă Gr. adj. *endo* within; N.L. n. *fausta* (host) *Calyptogena fausta;* N.L. n. fem. *endofausta*.
Properties: Intracellular thioautotrophic bacterial gill symbiont of *Calyptogena fausta* clams. Species affiliation defined molecularly by divergence (Figure 5 and [24]), by ≥95% average nucleotide identity genome-wide, and ≥98.7% sequence identity at rRNA, core metabolic, and housekeeping genes in the genome (*Calyptogena fausta* symbiont genome annotation under NCBI BioProject number PRJNA562081). These criteria place the *Ca.* Ruthia endofausta symbiont in the "group 2" vesicomyid symbiont clade from [24]. Furthermore, these criteria distinguish this species from the type species for the "group 2" genus *Candidatus* Ruthia, *Ca.* Ruthia magnifica (NCBI accession GCF_000015105.1), which is 66.8% identical genome wide and 97.9% identical at the 16S rRNA to *Ca.* Ruthia endofausta. As a member of the SUP-05 clade of sulfur-oxidizing bacteria, *Ca.* Ruthia endofausta is 42.7% and 95.8% identical at genome and 16S rRNA levels, respectively, to *Ca.* Thioglobus autotrophicus (NCBI accession GCF_001293165.1) in the closest-related genus.
Type host: *Calyptogena fausta* (Bivalvia: Heterodonta: Vesicomyidae)
Other hosts: None.
Type locality: Juan De Fuca Ridge, OR, USA.
Other localities: None.
Specimens deposited: None, as this bacterium is currently unculturable and is only known molecularly and microscopically. This is a tentative name proposed with the designation "*Candidatus*".

Name: *Candidatus* Vesicomyosocius endoextente sp. nov.
ĕn.dō.ĕx.tĕn.tĕ Gr. adj. *endo* within; N.L. n. *extenta* (host) *Calyptogena extenta*; N.L. n. masc. *endoextente*.
Properties: Intracellular thioautotrophic bacterial gill symbiont of *Calyptogena extenta* clams. Species affiliation defined molecularly by divergence (Figure 5 and [24]), by ≥95% average nucleotide identity genome-wide, and ≥98.7% sequence identity at rRNA, core metabolic, and housekeeping genes in the genome (*Calyptogena extenta* symbiont genome annotation under

NCBI BioProject number PRJNA562081). These criteria place the *Ca.* Vesicomyosocius endoextente symbiont in the "group 1" vesicomyid symbiont clade from [24]. Furthermore, these criteria distinguish this species from the type species for the "group 1" genus *Candidatus* Vesicomyosocius, *Ca.* Vesicomyosocius okutanii (NCBI accession GCF_000010405.1), which is 88.2% identical genome wide and 99.2% identical at the 16S rRNA to *Ca.* Vesicomyosocius endoextente. As a member of the SUP-05 clade of sulfur-oxidizing bacteria, *Ca.* Vesicomyosocius endoextente is 37.9% and 94.4% identical at genome and 16S rRNA levels, respectively, to *Ca.* Thioglobus autotrophicus (NCBI accession GCF_001293165.1) in the closest-related genus.

Type host: *Calyptogena extenta* (Bivalvia: Heterodonta: Vesicomyidae)

Other hosts: None.

Type locality: Monterey Canyon, CA 36-40.92N 122-7.21W

Other localities: None.

Specimens deposited: None, as this bacterium is currently unculturable and is only known molecularly and microscopically. This is a tentative name proposed with the designation "*Candidatus*".

Supporting References:

116. Rambaut A, Drummond AJ, Xie D, Baele G, Suchard MA. Posterior Summarization in Bayesian Phylogenetics Using Tracer 1.7. Susko E, editor. Syst Biol. 2018;67: 901–904. doi:10.1093/sysbio/syy032
117. Wakeley J, Aliacar N. Gene Genealogies in a Metapopulation. Genetics. 2001;159: 893–905.
118. Pannell JR, Charlesworth B. Effects of metapopulation processes on measures of genetic diversity. Philos Trans R Soc B Biol Sci. 2000;355: 1851–1864. doi:10.1098/rstb.2000.0740
119. Wakeley J. Metapopulation models for historical inference. Mol Ecol. 2004;13: 865–875. doi:10.1111/j.1365-294X.2004.02086.x
120. Komaki K, Ishikawa H. Genomic copy number of intracellular bacterial symbionts of aphids varies in response to developmental stage and morph of their host. Insect Biochem Mol Biol. 2000;30: 253–258. doi:doi: 10.1186/1741-7007-7-12
121. Wollenberg MS, Ruby EG. Population Structure of Vibrio fischeri within the Light Organs of Euprymna scolopes Squid from Two Oahu (Hawaii) Populations. Appl Environ Microbiol. 2009;75: 193–202. doi:10.1128/AEM.01792-08
122. Cavanaugh CM, McKiness ZP, Newton ILG, Stewart FJ. Marine Chemosynthetic Symbioses. In: Dworkin M, Falkow S, Rosenberg E, Schleifer K-H, Stackebrandt E, editors. The Prokaryotes. New York, NY: Springer New York; 2006. pp. 475–507. Available: http://link.springer.com/10.1007/0-387-30741-9_18
123. Klose J, Polz MF, Wagner M, Schimak MP, Gollner S, Bright M. Endosymbionts escape dead hydrothermal vent tubeworms to enrich the free-living population. Proc Natl Acad Sci.

2015;112: 11300–11305. doi:10.1073/pnas.1501160112
124. Duperron S, Quiles A, Szafranski KM, Léger N, Shillito B. Estimating Symbiont Abundances and Gill Surface Areas in Specimens of the Hydrothermal Vent Mussel Bathymodiolus puteoserpentis Maintained in Pressure Vessels. Front Mar Sci. 2016;3: 16. doi:10.3389/fmars.2016.00016
125. Sender R, Fuchs S, Milo R. Revised estimates for the number of human and bacteria cells in the body. 2016 [cited 22 Dec 2016]. Available: http://biorxiv.org/lookup/doi/10.1101/036103
126. Kaltenpoth M, Goettler W, Koehler S, Strohm E. Life cycle and population dynamics of a protective insect symbiont reveal severe bottlenecks during vertical transmission. Evol Ecol. 2010;24: 463–477. doi:10.1007/s10682-009-9319-z
127. Stephens WZ, Wiles TJ, Martinez ES, Jemielita M, Burns AR, Parthasarathy R, et al. Identification of population bottlenecks and colonization factors during assembly of bacterial communities within the zebrafish intestine. mBio. 2015;6: e01163-15. doi:10.1128/mBio.01163-15
128. Didelot X, Walker AS, Peto TE, Crook DW, Wilson DJ. Within-host evolution of bacterial pathogens. Nat Rev Microbiol. 2016;14: 150–162. doi:10.1038/nrmicro.2015.13
129. Mira A, Moran NA. Estimating Population Size and Transmission Bottlenecks in Maternally Transmitted Endosymbiotic Bacteria. Microb Ecol. 2002;44: 137–143. doi:10.1007/s00248-002-0012-9
130. Möhle M. A convergence theorem for markov chains arising in population genetics and the coalescent with selfing. Adv Appl Probab. 1998;30: 493–512. doi:10.1239/aap/1035228080
131. Möhle M, Notohara M. An extension of a convergence theorem for Markov chains arising in population genetics. J Appl Probab. 2016;53: 953–956. doi:10.1017/jpr.2016.54
132. Matuszewski S, Hildebrandt ME, Achaz G, Jensen JD. Coalescent Processes with Skewed Offspring Distributions and Nonequilibrium Demography. Genetics. 2018;208: 323–338. doi:10.1534/genetics.117.300499
133. Möhle M, Sagitov S. A Classification of Coalescent Processes for Haploid Exchangeable Population Models. Ann Probab. 2001;29: 1547–1562.
134. Eldon B, Wakeley J. Coalescence Times and FST Under a Skewed Offspring Distribution Among Individuals in a Population. Genetics. 2008;181: 615–629. doi:10.1534/genetics.108.094342
135. Yahara K, Didelot X, Jolley KA, Kobayashi I, Maiden MCJ, Sheppard SK, et al. The Landscape of Realized Homologous Recombination in Pathogenic Bacteria. Mol Biol Evol. 2016;33: 456–471. doi:10.1093/molbev/msv237
136. Dixit PD, Pang TY, Maslov S. Recombination-Driven Genome Evolution and Stability of Bacterial Species. Genetics. 2017;207: 281–295. doi:10.1534/genetics.117.300061
137. Kelleher J, Etheridge AM, McVean G. Efficient Coalescent Simulation and Genealogical Analysis for Large Sample Sizes. PLOS Comput Biol. 2016; 22.
138. Excoffier L, Foll M. fastsimcoal: a continuous-time coalescent simulator of genomic diversity under arbitrarily complex evolutionary scenarios. Bioinformatics. 2011;27: 1332–1334. doi:10.1093/bioinformatics/btr124
