# Supplementary material for: Horizontal transmission and recombination maintain forever young bacterial symbiont genomes
Source: PLoS Genet. 2020 Aug 25;16(8):e1008935. doi: 10.1371/journal.pgen.1008935 (PMC7473567; doi:10.1371/journal.pgen.1008935)
Supplement: Attachment — Submitted filename: r2r_ForeverYoung_PLoSGenetics.pdf [file pgen.1008935.s022.pdf]

Re (MS): PGENETICS-D-19-01871 (revised manuscript)
Russell, Pepper, Svedberg, Byrne, Ruelas Castillo, Vollmers, Beinart, and Corbett-Detig
Horizontal transmission and recombination maintain forever young bacterial symbiont genomes

Dear PLoS Genetics Editors,

Thank you for your and the reviewers' comments on our manuscript. We have carefully read, considered, and addressed all of the issues raised by the reviewers. These comments are printed in italics below, with our responses following each.

We especially appreciate the suggestions to infer divergence date estimates for hosts and symbionts and validate our recombination rate estimates. We think these new results have strengthened our argument that genome erosion can be prevented by ongoing symbiont horizontal transmission and recombination. Additionally, we appreciate the reviewers' suggestions regarding what background information was needed about these associations to better understand our rationale and results.

Thank you again for your comments. They have helped to improve the manuscript.

Shelbi Russell

Shelbi Russell, Ph.D.
University of California Santa Cruz
shelbilrussell@gmail.com

Notes from the Editors:
Your manuscript was fully evaluated at the editorial level and by three independent peer reviewers with complementary expertise. The reviewers appreciated the attention to an important problem, but raised some substantial and thoughtful concerns about the current manuscript. Based on the reviews, we will not be able to accept this version of the manuscript, but we would be willing to review again a much-revised version. We cannot, of course, promise publication at that time.
Should you decide to revise the manuscript for further consideration here, your revisions should address all the specific points made by each of the three reviewers. We will also require a detailed list of your responses to the review comments and a description of the changes you have made in the manuscript.

Reviewer's Responses to Questions

Comments to the Authors:

Reviewer #1: *This manuscript by Russell et al is a very interesting investigation of the evolution of bacterial endosymbionts of molluscs. The analyses are varied and complex, providing an overview of three different types of endosymbiotic association, and how they affect the evolution of the bacteria involved. The work has the potential to be an important contribution to the literature on fundamental processes in bacterial evolution, but the methodological novelty of the study means there is substantial clarification and validation required before the manuscript is ready for publication.*
The main points that need to be addressed are:

(1) The title makes reference to these bacteria maintaining "forever young" genomes. However, there is not much evidence for the long-term stability of the observed genotypes. S5 Table suggests there is not much diversity within each bacterial species (with very little diversity in the Calyptogena populations), and S7 Fig suggests much of this is likely to be due to be the result of a small number of long branches. Using the most relevant molecular clock data available, the authors should estimate (A) the time to the most recent common ancestor of the sequenced bacteria (eliminating the diversification through recombination as the coalscent analysis should allow, but it should not make much difference for Calyptogena), and (B) compare this to the estimated times at which the host species diverged. This should estimate the duration over which the symbiosis can be analysed. An alternative hypothesis to that proposed by the authors is that the ability for horizontal acquisition of these bacteria by hosts makes it possible for environmental bacteria to invade the niche and displace the resident symbionts (the "symbiont replacement" mentioned in the Introduction), explaining the observed data (including any most recent common ancestors that substantially post-date the divergence of hosts) without the genomes being "forever young". Rather, this would support the model in which the genomes of the symbionts degrade, and are intermittently replaced by new species from the environment.

Thank you for bringing to our attention that we had not adequately conveyed the antiquity of these associations, which we know to be true based upon the fossil record for the host and 16S rRNA phylogenies for the symbiont. However, we agree that more direct and quantifiable evidence about the age of these associations would make our findings more compelling. So, we took your advice and estimated divergence dates for host mitochondrial and symbiont genomes. We found that the thioautotrophic Bathymodiolus symbionts are monophyletic, diverged at a similar time as their host Bathymodiolus clade (~100-150 mya), and are around 220 million years diverged from their closest free living relative. Similarly, the S. velum symbiont and its closest relative, the S. elarraichensis symbiont have co-speciated with their hosts who diverged 129 million years ago, and are up to 942 million years diverged from their free-living relatives. Although, this part of the gammaproteobacterial tree is

undersampled, so there may be closer extant relatives to the S. velum and S. elarraichensis symbionts. And, while you are absolutely right, there have likely been replacement events in some hosts over time, these too appear to be ancient. We present these results in a new figure, Fig. 5, and in the following sections of the manuscript:

> Results and Discussion (lines 278-308): “Divergence date estimates for hosts and symbionts indicate that the observed patterns in symbiont genome structure have been maintained over many millions of years (Fig. 5, S8 Fig., and S10-12 Tables). Similar to prior work [21], we estimated that the vesicomyid bivalves evolved from their non-symbiotic ancestors around 73 million years ago (mya) (95% highest posterior density (HPD) = 62.59 - 76.70 my; Fig. 5B). We estimated a similar divergence date for the vesicomyids’ monophyletic symbionts of around 84 my (95% CI = 42.37 - 165.09 my; Fig. 5A), which is remarkable given that their loss of DNA repair genes and reduced selection efficacy has likely increased their substitution rate (and may have been accounted for by using a relaxed local molecular clock). Interestingly, the clade of gammaproteobacteria that contains the vesicomyid symbionts, termed the SUP05 clade, also contains the thiotrophic Bathymodiolus symbionts and free-living bacteria with genomes in the range of 1.17-1.71 Mb (Fig. 5A), from which the vesicomyid symbionts are approximately 220 my (95% CI = 127 - 381my) diverged. This indicates that the vesicomyid symbiont genomes have eroded 58% at most, depending on the symbiont lineage and the ancestral state. Thus, over tens of millions of years of host association, the vesicomyid symbiont genomes have exhibited high degrees of stasis and have only degraded moderately (e.g., in vesicomyid symbionts with 1 Mb vs. 1.2 Mb genomes).
>
> The solemyids present a similar, but more complicated situation, likely owing to their antiquity, as the hosts first appeared in the fossil record more than 400 mya [22], when the ocean basins had much different connectivity [57]. While host-switching and novel symbiont acquisition have certainly occurred in Solemyidae, and our data indicates such an event may have happened after S. velum and S. pervernicosa diverged (Fig. 5A), it may be relatively rare across geological time. Whole genome mitochondrial and symbiont phylogenies indicate that the North Atlantic Solemya species S. velum and S. elarraichensis are sisters that diverged around 129 mya (95% HPD = 124 - 152 my; Fig. 5B), and their symbionts likely co-speciated with them around the same time (170 mya, 95% CI = 89 - 325 my; Fig. 5A). Divergence and subsequent speciation may have been due to the opening of the Atlantic, which occurred contemporaneously (180-200 mya; [58]). Thus, the vertically transmitted S. velum and S. elarriachensis symbionts have maintained genomes similar in size to their free-living relatives over hundreds of millions of years of host association.”
>
> Materials and Methods: see the new “Divergence dating” section in “2. Genome analyses” on lines 544-602. Additional details were also added to S1 Supplemental Text.

Regarding the lack of variation in these populations, it is likely that this is caused by frequent local extinction and recolonization events, as this pattern is also repeated at the metapopulation level in S. velum (see Russell et al. 2017). Geologically, this is reasonable because hydrothermal vents are ephemerial and coastal reducing sediments can be disturbed by storms.

(2) The authors state "the other [non-vesicomyid] symbiont genomes are highly structurally dynamic". However, Figure 4E demonstrates that these non-vesicomyid symbionts are polyphyletic – it is not surprising that there are many changes distinguishing the aligned pairs. The authors need to provide evidence that these representatives have had an endosymbiont lifestyle over the full period of their divergence (are the related species all endosymbionts?), or make clear they have become endosymbionts in parallel, and the genetic divergence has occurred outside of these hosts. The rest of the paragraph is confusing, not least owing to a lack of specificity ("It is", "these groups"). The authors state that "This could proceed by iteratively locking deletions (initially caused by recombination… With recombination events occurring so rarely in these endosymbiont genomes, natural selection is unable to efficiently purge deleterious deletions. Inversions, which can be highly mutagenic by inverting the translated strand, inducing replication-transcription machinery collisions [33], may be nearly absent due to their high fitness costs." – does recombination increase or decrease the rate at which deletions accumulate, and is selection weak (enabling deletions) or strong (preventing inversions)? Additionally, when stating "This is consistent with a combination of free-living and host associated periods", they should specify whether this refers to the species current lifestyles (which would conflict with the description of these bacteria as obligate symbionts in the abstract, though this phrase is not repeated in the main paper), or over the history of the observed species.

Thank you for raising these important points and indicating where we could improve our clarity in discussing how recombination impacts bacterial genome structure. While the thiotrophic Bathymodiolus symbionts are quite closely related, and according to our newly inferred whole genome phylogenies (described above to address your first comment) are monophyletic, you are absolutely correct that the S. velum and S. pervernicosa symbionts are polyphyletic. They are actually fairly distantly related, although they do reside in the same larger clade of symbionts and free-living sulfur-oxidizing taxa. Fortunately, a draft genome is available for the sister species of the S. velum symbiont, the S. elarraichensis symbiont, which permits us to look at local synteny (along a maximum contig length of 38 kb) at much shorter time-scales. Interestingly, despite there being ~129 million years of divergence between the S. velum and S. elarraichensis symbionts, their genomes exhibit complete synteny over at least tens of kb, although there are many large insertions and deletions. We have updated the manuscript to convey these results and clarify our description of the deletion process with a new Figure S9 and the following paragraphs in the Results and Discussion (lines 234-265):

> "Genome structure stasis is thought to be another hallmark of canonical endosymbiont genome evolution, and many terrestrial endosymbioses have reported static, degenerate genomes [1]. Whole genome alignments reveal that the vesicomyid symbiont genomes are highly syntenic, with few rearrangements, insertions, or deletions; whereas the other symbiont genomes are far more structurally dynamic (Fig. 4 and S7 Fig.). Given the role of recombination in altering bacterial genome structure ([50]; Fig. 4A) and the signal of linked selected sites in the vesicomyid symbionts (Fig. 3), recombinational processes may partially underlie genome erosion. This could proceed in the following way: first, a rare recombination event induces a deletion that drifts to high frequency in the within-host population. With homologous recombination events occurring so rarely in these endosymbiont genomes, natural selection would be unable to efficiently purge deleterious deletions. Although, inversions, which can be highly mutagenic by inverting the translated strand, inducing replication-transcription machinery collisions [53], are nearly absent potentially due to their high fitness costs. If symbionts with chromosomes bearing the recombination-induced deletion are exclusively transmitted to offspring during vertical transmission, then the deletion would be fixed in all subsequent

symbionts in that host lineage. Multiple instances of this process would incrementally reduce the size of the symbiont genome.

In contrast, the solemyid symbiont genomes exhibit increasing degrees of structural dynamics with increasing divergence time. Highly divergent solemyid symbionts, such as the S. velum and S. pervernicosa symbionts, exhibit genomes that are as structurally dynamic as strictly horizontally transmitted associations (Fig. 4B vs C). However, over shorter time scales, such as the duration of time since the S. velum and S. elarraichensis symbionts diverged (discussed below), structural changes appear to be dominated by insertions and deletions (indels; confirmed for 30 Kb segments of the S. elarrachensis symbiont draft genome in S7 Fig.). Rapidly evolving indels have also been reported at the species level for S. velum [15]. Potentially underlying indel dynamics, we found that the solemyid symbionts have far more mobile genetic elements than any of the other symbiont genomes (S8 Table). High mobile element content is consistent with a combination of environmentally-exposed and host-associated periods [54], and mirrors the early stages of symbiosis [19,20], as well as the early stages of eukaryotic asexuality [55]. The mobile elements exhibit homology to different environmental bacteria, implying many independent insertion events (S9 Table). "

The above statement should also address your concern about the "free-living" statement. This was meant to indicate environmentally-exposed life stages, rather than the ability to divide outside of the host (which is often the interpretation of "obligacy" for a bacterium).

*(3) The authors refer to recombination repeatedly, but never specify whether they mean homologous recombination (cross over or gene conversion), horizontal gene transfer, or mobile elements. They should make clear which they mean in each analysis. In the S2 Supporting Text, they refer to "When a recombination event occurs on a genealogy, it creates an additional lineage to the right of the recombination breakpoint." – this sounds like a crossover event, which is not a typical form of recombination in bacteria. The text does not specify the interpretation of rho, rho*l or r/m sufficiently. Most bacterial geneticists would interpret r/m as being the ratio of single nucleotide variants occurring through recombination to those occurring through point mutation (e.g. Feil et al 1999 Mol. Biol. Evol. 16(11):1496–1502), yet the r/m for C. fausta is 3625 (it is rarely over 10 for any bacterial species except the most rapidly recombining), despite it having a low rate of recombination and only 815 segregating sites. It seems likely the analysis is prone to false positive inferences of recombination – the calculated rates are high, and even bacteria lacking recA were found to undergo recombination. The authors should report the estimated rate of recombination for an analogous analysis of the mitochondrial DNA sequences to demonstrate the method is robust to false positives – perhaps additionally the method could be validated against datasets where r/m has been calculated by alternative methods?*

Thank you for these very helpful comments. We agree that we need to be more precise in our definitions and descriptions of recombination, recombination rate, and recombination tract length, and that a validation of our method would be valuable. We present several additions to the text that address these issues below.

Whenever we refer to recombination, we mean homologous recombination, whereas we say horizontal gene transfer when referring to events, such as non-homologous recombination, that result in the incorporation of non-homologous DNA. However, we see how this can be confusing and needs to be

explicitly defined, and so we have done so throughout the text. We have also fully defined rho and rho*l in the Methods with the following added text on lines 615-618:

> “Here, we define the population-scaled per-base pair recombination rate, rho, to be equal to two times the effective population size times the per-base pair rate of gene conversion (rho=2*N_er). The recombination tract length, l, is defined as the length of the gene conversion segment in base pairs.“

Regarding the section referenced from S2 Supporting Text, this is an accurate description of how we modeled bacterial recombination events as essentially gene conversion events. Coalescent models generally make genetic modifications each generation from left to right along the chromosome, which is why we say “it creates an additional lineage to the right of the recombination breakpoint”. Indeed, additional lineages are created to the right of both breakpoints. But, we see how this phrasing could be misleading, as it does sound like crossing over. To address this, we have altered the section quoted above so it now reads:

> “When a gene conversion event occurs on a genealogy, it creates an additional lineage within the converted segment to the right and to the left of the recombination breakpoints.”

In regards to your comments about r/m, we realized that we have conflated two related, but fundamentally distinct terms. Specifically, we estimate the impact of recombination as rho*l/theta (as in de Maio and Wilson 2015), which we inadvertently described as equivalent to R/M originally (and has been removed from the text). rho*l/theta is typically larger than R/M and our results are consistent with some previous estimates. We have corrected this in the text and supplemental table.
Additionally, we now apply our approach to examine a similar dataset from Bacillus cereus (Didelot et al. 2010). In doing this, we find that our estimate is intermediate to many that have been produced previously using related approaches. Importantly, this suggests that our approach is not prone to falsely inflating estimates of the impact of recombination. We also provide some qualified speculative discussion of the possible reasons why recombination might be more prevalent than in ours than in many bacterial datasets that have been studied to date. The relevant sections now read:

> Results and Discussion (lines 207-209): “For all three symbiont taxa, recombination has a larger impact on genome evolution than mutation (estimated by rho*l/theta in S4 Table), in part due to the relatively low estimates of theta in the symbiont populations.”
>
> Methods (lines 652-660): “Method validation on existing datasets:
> In light of the relatively high recombination rates that we estimate, it is valuable to confirm that our approach for estimating rho and theta performs as expected. We therefore applied our method to the Bacillus cereus dataset [113] that has been studied in similar contexts using several related approaches [111,113,114]. In prior work with this dataset, estimates of the total impact of recombination, rho*l/theta, have varied somewhat, from approximately 3.7 [111] to 35.9 [113] and 229 [114]. Using our method, we obtain a value intermediate to these at 17.1, which indicates a moderate impact of recombination on genome evolution. This result suggests that our method is reliable and does not substantially inflate recombination rate estimates.”

Minor criticisms:
• *S1 Table should be a spreadsheet with accession numbers for the included samples*

S1 Table now contains a column for the accession numbers, which will be added when the data is submitted to NCBI directly before publication. This spreadsheet is uploaded as an excel file.

• *Figure 3B needs labelled scale bars – the trees should be presented more clearly, with leaf tip names clearly readable. Data from S7 Fig should be included as well.*

We have moved the scale bars from the figure legend into the figure and added the data from S7 Fig. While we want to comply with your request for leaf tip labels, we do not see why it is necessary and think it actually distracts from highlighting the distributions of branch lengths (which is the point of the figure).

• *Figure 4E should be a separate, enlarged figure, with leaf tip names clearly readable*

Thank you for this suggestion. We came to the same conclusion when replacing the tree with the symbiont and mitochondrial whole genome dated phylogeny. These images now comprise Fig. 5.

• *The supplementary tables need more detailed legends, particularly S4 Table, S6-S8 Tables*

We have expanded all four of these table legends to include extra details about the table contents and how it was generated.

• *The authors need to explain how the p values on Pg. 9 Ln. 164-167 were calculated*

Thank you for catching that we omitted this detail. We have corrected the text to now read:

> "...with dN/dS values of 0.14, 0.096, 0.083 for vesicomyid, solemyid and bathymodiolin genomes, respectively (pairwise Wilcoxon test p-values: bathymodiolin-vesicomyid p=4.60e-14, solemyid-vesicomyid p= 1.02e-11, and bathymodiolin-solemyid p= 0.0365)..."

• *Why were genes with extreme dN/dS ratios excluded, rather than genes with small numbers of mutations? "Wilcox test" should be "Wilcoxon test".*

Genes with extreme dS values were removed because estimates of dN/dS are unreliable for those values. Saturated mutations make ratios meaningless and small numbers of mutations have low power. We did mention this in the Materials and Methods section with the following statement: "We excluded all comparisons for which dS < 0.05 or dS > 2, as values that exceed this range are often thought to yield unreliable estimates of rates of molecular evolution". However, to clarify further, we have amended this statement to add (lines 539-541):

> "...as values that exceed this range are often thought to yield unreliable estimates of rates of molecular evolution due to low statistical power and saturated substitutions, respectively."

Thank you for pointing out the typo. We have made the suggested corrections to the manuscript.

• *The four gamete test should be more thoroughly explained.*

Yes, we agree that more explanation is needed. We have added the following section to the Material and Methods section to describe how we performed the 4-gamete test (lines 483-486):

> "Then we used the aligned SNP data to calculate Waterson's theta [87], pi [88], and the proportion of pairwise sites where all 4-gametes, i.e., all pairwise combinations of alleles, are represented. We then binned the 4-gamete sites by the distances between alleles, with bins at 1e1, 1e2, 1e3, 1e4, 1e5, and 1e6 bp, for model fitting (described below)."

• *"Nanopore" and "Illumina" are sometimes not capitalized. There are contractions in the main and supplementary text (e.g. "that's").*

Thank you for pointing out these typos. We have corrected them in the manuscript.

Reviewer #2: *Summary:*
This study investigates the genome evolution of gammaproteobacterial symbionts from three groups of bivalves and provides an interesting symbiont study system, with the three host groups exhibiting different modes of symbiont transmission: horizontal, vertical and mixed mode transmission. Surprisingly, one symbiont group with reduced genomes has maintained a relatively stable genome size that is likely due to a moderate genetic recombination rate compared to others like insect symbionts with severely reduced genomes. This is a great study, but there are several aspects that need clarification and the authors have focused on the idea that the symbionts can maintain stable genome size indefinitely without much evidence or a discussion of other possible outcomes.

Major Comments:
Although using "forever young" in the title is catchy, this may not be true as stated in lines 186 – 187. At this moment in time, vesicomyid symbionts may be able to slow genome degradation and maintain an intermediate genome size, but the processes of Muller's ratchet are still at work and continued genome reduction/degradation is a likely outcome. In which case, lines 206 – 207 also need to be rephrased, especially the term "indefinitely".

These are really good points that we think some of our added analyses help to address. In response to Reviewer 1's first comment, we inferred dated whole genome phylogenies for both the hosts and symbionts. These show that the vesicomyids have co-speciated with their symbionts over at least 73 million years. We take this long period of divergence and consistency in genome size to indicate that degradation has definitely been stalled. While we cannot say what the future holds for these associations, as population dynamics could change so that loss of coding content is permitted, we maintain that the exiting evidence suggests that horizontal transmission and recombination rates have been high enough to prevent this so far. To clarify this point in the text, we have added/changed the following paragraph on lines 278-294 of the Results and Discussion:

"Divergence date estimates for hosts and symbionts indicate that the observed patterns in symbiont genome structure have been maintained over many millions of years (Fig. 5, S8 Fig., and S10-12 Tables). Similar to prior work [21], we estimated that the vesicomyid bivalves evolved from their non-symbiotic ancestors around 73 million years ago (mya) (95% highest posterior density (HPD) = 62.59 - 76.70 my; Fig. 5B). We estimated a similar divergence date for the vesicomyids' monophyletic symbionts of around 84 my (95% CI = 42.37 - 165.09 my; Fig. 5A), which is remarkable given that their loss of DNA repair genes and reduced selection efficacy has likely increased their substitution rate (and may have been accounted for by using a relaxed local molecular clock). Interestingly, the clade of gammaproteobacteria that contains the vesicomyid symbionts, termed the SUP05 clade, also contains the thiotrophic Bathymodiolus symbionts and free-living bacteria with genomes in the range of 1.17-1.71 Mb (Fig. 5A), from which the vesicomyid symbionts are approximately 220 my (95% CI = 127 - 381my) diverged. This indicates that the vesicomyid symbiont genomes have eroded 58% at most, depending on the symbiont lineage and the ancestral state. Thus, over tens of millions of years of host association, the vesicomyid symbiont genomes have exhibited high degrees of stasis and have only degraded moderately (e.g., in vesicomyid symbionts with 1 Mb vs. 1.2 Mb genomes)."

We have also worked to estimate a minimum age of continuous association in the solemyids. While the *S. pervernicosa* and *S. velum* symbionts are paraphyletic, we do have the genomes for a clade in which hosts and symbionts are both monophyletic: *S. elarraichensis* and *S. velum*. Including the *S. elarraichensis* dataset (from Russell et al. 2017) in the divergence dating analysis revealed that these bacteria have been host-associated for at least 129 million years. Since these genomes are truly the size of their free-living relatives after this extremely long period of association, we think this is sufficient evidence to justify our title. These results have also been discussed in the Results and Discussion section, below the paragraph quoted above (lines 296-308) (and described more above in response to Reviewer 1).

I found it hard to follow the population genetics discussion without having more ecological context of the bivalve systems. Please include a short description of the transmission modes (how many symbionts are transferred during vertical transmission, how host switching occurs, how the hosts gain symbionts from the environment, are the symbionts obligate intracellular bacteria or facultative, etc.).

This is a great idea. We've added the following information to the end of the last paragraph of the Introduction to more completely describe the ecology of these associations (lines 124-134):

"...Hosts are nearly completely dependent on symbiont metabolism [38], and the solemyids have lost the majority of their digestive tracts in response [39]. These associations appear to be obligate for the symbionts as well as the hosts because either the symbionts have never been found living independently, e.g., solemyid [31] and vesicomyid symbionts [40], or they have only been found in the host and surrounding environment, e.g., bathymodiolin symbionts [27,41,42]. Both the vesicomyids and solemyids transmit symbionts to their offspring through allocating tens to hundreds of symbiont cells to their broadcast spawned oocytes [31,33]. While the mechanism of horizontal, host-to-host transfer has not been identified in the Bathymodiolus or solemyid symbionts, signatures of rampant horizontal transmission are evident in the population genetics of both of these groups [15,27,28] and the developmental biology of Bathymodiolus [29]."

An "organelle-like state" is mentioned throughout the paper, but this term is highly debated. What is your definition of organelle-like?

Indeed, you are correct that what constitutes an organelle is highly debated. That's why we tried to tone it down by saying organelle-like. But, you are absolutely correct that we should explicitly define what we mean by this term. We've added details to the following sentence on lines 85-88 in the Introduction to address this:

> "...Ultimately, this process is thought to result in an organelle-like genome that is a fraction of the size of its free-living ancestors and has relegated many of its core cellular functions (e.g., DNA replication and repair, cell wall synthesis, etc.) to the host or lost them entirely."

Line 685 (Figure 1): It's interesting that the vesicomyid symbiont and mitochondrial phylogenies are discordant since those symbionts are vertically transmitted. Can you explain more about why there are "sufficient amounts of horizontal transmission" occurring in vesicomyid symbionts? Also, please explain the difference between host-switching and horizontal transmission in vesicomyid symbionts. Can these symbionts be found in the environment (e.g. free-living) or do they become transferred directly from other non-related hosts?

This is a great question that deserves to be addressed in the text. Theory has shown (e.g., Brandvain et al. 2011) that the linkage between organellar genomes, which underlies genealogical concordance, breaks down with very low amounts of horizontal transmission. So, basically, seeing concordance is indicative of vertical transmission, but seeing discordance only is only indicative of a departure from strict vertical transmission. It doesn't tell you anything about the rate of horizontal transmission. We have added the following explanation at the beginning of the Results and Discussion (lines 152-163):

> "Comparative analyses of host mitochondrial and endosymbiont genome genealogies show strong evidence for horizontal transmission in all six populations (Fig. 1C). Despite this apparent similarity in transmission mode, the vesicomyid endosymbiont genomes are approximately one half the size of the solemyid or mytilid endosymbiont genomes, which themselves are approximately consistent with their free-living ancestors. Nonetheless, at 1-1.2 Mb, the partially degraded vesicomyid endosymbiont genomes are still ten times larger than the smallest terrestrial endosymbionts [1]. The genealogical discordance shown in Figure 1C indicates that horizontal transmission has occurred in the histories of these populations, however, it does not suggest how much because concordance is eroded even by exceedingly low rates of horizontal transmission [43], saturating the signal genealogies can provide. Thus, a finer scaled exploration of symbiont genetic diversity was necessary to characterize the population-level processes influencing genome erosion."

Another aspect that I found interesting is that the vesicomyid symbionts have the smallest genomes of the bivalve symbionts but evolved from their free-living ancestors much later (66 mya) than the solemyid symbionts (400 mya). Therefore, vesicomyid symbiont genome reduction occurred relatively quickly compared to the solemyid symbionts. Also, the solemyid symbionts seem to have evolved from free living ancestors multiple times whereas the vesicomyid symbionts arose only once. Are these differences due strictly to transmission modes or are there other factors involved?

These are great observations. There appear to be a few things going on that work together to produce the patterns you described. First, the clade that the vesicomyid symbionts are in, the Sup05 clade, which also contains the thiotrophic Bathymodiolus symbionts and free living taxa, has smaller than average genomes. So, it is likely that the symbionts entered the association with smaller genomes than the average for sulfur-oxidizing bacteria. Second, the solemyids present an interesting case likely due to their age. The fossil record indicates that the solemyids have been living in reducing sediments, probably employing symbiont-mediated chemoautotrophy, since their evolution ~400 mya. If you look at the pattern of solemyid symbiont distribution with 16S rRNA sequences (see Russell et al. 2017), for which we have more species represented, it appears that the symbionts are related within a general locality such as the North Atlantic, North Pacific, etc. So, it seems that each group of solemyids switched symbionts upon arriving and speciating in each ocean basin. We have tried to work some of this information into the discussion about symbiont and host divergence dates at the end of the Results and Discussion section (lines 296-308).

Also according to the symbiont phylogenetic tree, the solemyid symbionts evolved multiple times from free living ancestors and the mytilid symbionts evolved at least twice. Have these hosts replaced symbionts multiple times with free-living bacteria that eventually became new symbionts and thus, a range of symbiont genome sizes exist (at least in the solemyid symbionts)? This has happened in other symbiosis systems like mealybugs (Husnik et al. 2016), lice (Smith et al. 2013) and the protist, Euplotes (Boscaro et al. 2017) so it seems possible that these host groups also replaced the symbionts when deleterious mutations were fixed in symbiont populations, especially mutations in carbon fixation pathways.

Yes, symbiont replacement is definitely a possibility. As we discussed above, we think this is what has occurred in different groups of solemyids, and we have tried to make this idea clear in the text. Although, we would argue that perhaps replacement in these cases may have been opportunistic, or due to locally-adapted strains out-competing the resident symbiont, rather than a consequence of genome erosion. But, of course these do not need to be mutually exclusive.

In the case of the mytilid symbionts, the thiotrophic symbionts appear to be monophyletic (and related to the vesicomyid symbionts), whereas the methanotrophic symbionts are in a separate, more distantly related clade. We've added the following sentences to the Results and Discussion and the Figure 5 legend describing host and symbiont divergence-dated trees to make this clear:

> Results and Discussion (lines 286-289): "...the clade of gammaproteobacteria that contains the vesicomyid symbionts, termed the SUP05 clade, also contains the thiotrophic Bathymodiolus symbionts and free-living bacteria with genomes in the range of 1.17-1.71 Mb (Fig. 5A)..."
>
> Figure 5 legend: "...In both phylogenies, members of vesicomyid, solemyid, and bathymodiolin (both thioautotrophic and methanotrophic) associations are colored yellow, green, and blue, respectively..."

Although a few examples of other symbiosis systems are included, a quick summary about the processes that drive genome reduction in other symbionts (e.g. insect symbionts) compared to those in the bivalve symbionts is needed. What are some differences and/or similarities between the systems (selection, drift, transmission, age of the symbiosis, etc.)? This would be a nice place to discuss why the vesicomyid symbionts have

maintained a stable genome size whereas other systems have severely reduced symbionts. What are the processes involved and why would the vesicomyid symbionts have a different outcome?

Unless I have misunderstood your comment, we do not think there are intrinsic differences in the processes that drive genome reduction in these groups. Reducing symbiont population size and imposing transmission bottlenecks leads to an evolutionary trajectory dominated by drift, which ultimately leads to genome erosion, in all of these associations. We think this is explained pretty thoroughly in the second paragraph of the Introduction. The distinction we are making here for these marine associations is that symbiont population size is not restricted as much as one might assume by an intracellular localization and transmission through host gametes, leading to gene flow and recombination that prevents genome decay. We have tried to highlight this a bit more clearly by adding the following sentence in the Conclusion paragraph on lines 325-326:

> "Amazingly, we found that symbiont gene flow between hosts is ongoing in one of the most intimate marine associations, the vertically transmitted vesicomyids."

Minor comments:
Please introduce the study system in the abstract since it is not mentioned until the last introduction paragraph.

The following sentence has been updated in the Abstract to briefly describe the symbionts and hosts:

> "By sampling marine chemosynthetic bacterial endosymbionts of bivalves that range from primarily vertical to strictly horizontal transmission, we tested this canonical theory. "

(Line 33) Eukaryotes have not existed for the "history of life on Earth"

Thanks for letting us know this was not interpreted the same way we meant it. We meant, over the period of history they have existed for, but understand how this is confusing/misleading. We've edited the sentence to remove that bit, as it was not really needed.

(Line 75 – 76) There are known marine symbiont genomes less than 1 Mb: bacterium AB1 in the marine invertebrate, Bugula neritina (Miller et al. 2016); Spiroplasma holothuricola in sea cucumbers (He et al. 2018); alphaproteobacteria in the marine protists, diplonemids (George et al. 2019)

Thank you for bringing these genomes to our attention; we had not yet seen the 2020 Current Bio article by George et al.. Regarding the other genomes: we had missed that bacterium AB1's genome was closed, but had also discluded it because of Miller's description of it as a "transient" association. Similarly, it was not clear from our reading of He et al. 2018 that Candidatus Spiroplasma holothuricola is known to be an obligate symbiont (although it is clearly the leading hypothesis). We wanted to be conservative in what we were considering an obligate symbiont. However, given the convergently reduced diplonemid symbiont genomes in George et al. 2020, we were obviously too conservative. We have changed the following sentence in the Introduction to better reflect the distribution of symbiont genome sizes in marine hosts (lines 92-94):

> "In particular, most known marine obligate endosymbionts' genomes are at least one megabase in size and contain diverse coding content [11–17], although reduced representatives have recently been found [18]. "

(Figure 2) Switch Panel C and D labels so that the labels go in alphabetical order.

The labels are arranged in the order they are mentioned in the text. If we switch them, then they will be mentioned out of order (D before C). And, if we rearrange them so the panels are in alphabetical order, the panels will not fit in a rectangle. Since the other two reviewers did not have an issue with the panel order, and we do not have a remedy for the aforementioned problems associated with relabeling, we are leaving the figure as-is.

Reviewer #3: *The authors ask an important and timely question about how the long-term stability of host-symbiont associations influences the symbiont genome evolution. They address the question by comparing some genomic properties of chemosynthetic gill-inhabiting bacterial symbionts in seven species representing three families of bivalves, with different modes of the symbiont transmission.*

The authors have generated substantial amounts of novel quality data. I feel that the presented work has the potential of adding substantially to our understanding of symbioses of these bivalves, and symbioses more generally. However, I have several major concerns.

The presented data focus on seven bivalve species from three families with different symbiont transmission modes. Considering this, I feel that the author's claim of "testing the canonical theory" "by sampling marine endosymbionts that range from primarily vertical to strictly horizontal transmission", and that "These results validate long-standing but untested theory and demand a reinterpretation of the vast diversity of symbioses..." is an overstatement. I argue that major evolutionary theories relevant to a wide range of systems cannot be adequately tested using only three independent data points (=families). While the theoretical background provided in the Introduction is solid and relevant, I strongly recommend that the authors shift the focus of this study to what the study actually is: a comparative description of symbioses in three families of bivalves.

This is a fair opinion of our work and study system. Indeed, we are only studying three families of hosts and one type of symbiont, albeit in a set of fairly diverse chemoautotrophic clades at the base of Gammaproteobacteria. However, as there are few population genomic investigations of microbial symbionts, let alone ones representing multiple independent associations, with multiple species sampled that have characterized transmission modes, we feel that this is a relatively broad body of work that justifies extrapolation to other groups. Furthermore, we do believe that our work is broadly relevant because the population genetic principles underlying symbiont genome dynamics are the same across all associations. Host and symbiont biology dictate which of these principles dominate and the precise parameter values, *e.g.*, when horizontal transmission enables gene flow. As there are other intracellular obligate associations in the marine environment that exhibit similar biologies, it is likely that the observations we made here will be relevant to those associations. This is why we discuss the marine environmental component in the following sentence in the Introduction: “Symbioses in marine environments exhibit significantly more horizontal transmission between hosts than those in terrestrial environments [25], suggesting that symbiont gene flow between hosts may prevent genome decay by enabling high rates of homologous recombination, efficient natural selection, and the maintenance of highly diverse genome contents”.

To ensure the reader is aware that our study is making broad claims by extrapolation from only three families of hosts, we have added the following qualification to the Conclusion (lines 321-324):

> "Although we have only investigated three independently evolved associations, we see this system serving as a microcosm for marine associations more generally, as many other associations exhibit similar biologies (e.g., intracellular, autotrophic, broadcast-spawning, etc. [36]) and all are governed by the same population genetic principles."

In an apparent attempt to make the paper appear relevant to a broad audience, the authors hid the information of what they actually studied. I found it striking that bivalves - the animal group that was studied - is not mentioned in the title, abstract, or author summary, not even once! Furthermore, the authors provided very little introductory background information for these bivalves or their symbionts [lines 100-102]. As the only reference to support the statement that the three bivalve families differ in their modes of symbiont transmission, they provided a meta-analysis of >500 host-microbe symbioses, and some of the results of the current study. More information about the nature and old age of these symbioses is provided at the end of the Discussion - not a place where readers typically look early on. Without that background information or experience with bivalve symbioses, I have found it difficult to understand what the authors have done and why, and what the results mean. Some of the key questions that the Introduction should address are:

** What is known about bivalve symbioses? What are their functions and localizations? How specific are they?*
** Are the focal families obligately associated with symbionts?*
** In their gills, do bivalves host single symbiont strains, or more complex multi-strain or multi-species microbiota?*
** How these symbionts transmit - what did we know before the current study?*
** What has been known about the functions of these symbionts?*
In my opinion, the authors need to develop the second half of the Introduction around these questions, explaining how their presented work fills gaps in our existing knowledge.

We have added as much background for these associations as was possible while keeping the Introduction succinct and focused. For more information about these fascinating associations, we have provided references to reviews that provide more information. We have also added the primary references for the symbiont transmission modes. The following paragraphs are now at the end of the Introduction and should address all of these issues (lines 115-148):

> "To determine which evolutionary forces prevent marine endosymbiont genomes from degrading despite host restriction, we leveraged both population and comparative genomics of six marine bacterial-animal symbioses from three host taxa that exhibit modes of transmission across the spectrum from strict horizontal transmission (Bathymodiolus mytilid mussels) [27–29], to mixed mode transmission (solemyid bivalves) [15,30–32], to nearly strict vertical transmission (vesicomyid clams) [23,33–35] (see Fig. 1A-B and S1-S3 Tables). Each of these three groups evolved independently to obligately host either a single intracellular gammaproteobacterial symbiont 16S rRNA phylotype within their gill cells or two or more phylotypes, in the case of some mussels [36,37]. These symbionts provide chemosynthetic carbon fixation, either through sulfide or methane-oxidation, to nutritionally support the association [25]. Hosts are nearly completely dependent on symbiont metabolism [38], and the solemyids have lost the majority of their digestive tracts in response [39]. These associations appear to be obligate for the symbionts as well as the hosts because either the symbionts have never been found living independently, e.g., solemyid [31] and vesicomyid symbionts [40], or

they have only been found in the host and surrounding environment, e.g., bathymodiolin symbionts [27,41,42]. Both the vesicomyids and solemyids transmit symbionts to their offspring through allocating tens to hundreds of symbiont cells to their broadcast spawned oocytes [31,33]. While the mechanism of horizontal, host-to-host transfer has not been identified in the Bathymodiolus or solemyid symbionts, signatures of rampant horizontal transmission are evident in the population genetics of both of these groups [15,27,28] and the developmental biology of Bathymodiolus [29].

This group of marine symbioses presents an ideal system in which to test the impact of transmission mode and homologous recombination rate on bacterial symbiont genome evolution. The wealth of information known about how the vesicomyid, solemyid, and bathymodiolin symbioses function and evolve makes this a powerful evolutionary model. Furthermore, the similarity of these associations to other invertebrate-bacterial associations in the marine environment, e.g., in mode of reproduction (broadcast spawning), phylogeny (Mollusca-Gammaproteobacteria), immunology (innate), ancestral feeding type (filter or deposit feeders), symbiosis function (nutritional)), makes this an ideal microcosm for understanding how symbiont population genetics impact the process of symbiont genome degradation. Here, we use this model study system and a powerful population genomic approach (described in S1 Fig and S1-S3 Supporting Text), to show that homologous recombination is ongoing even in the most strictly vertically transmitted associations and may enable the maintenance of large and intermediate genome sizes indefinitely."

Additionally, we added information about the host and symbiont taxa to the Abstract and Author Summary.

Another major weakness of the study is the way information is organized in the article. I struggled to understand what exactly the authors did, how they did it, what was the reasoning, and which of the findings are new. I provided one example in the previous paragraph: the authors state that they have compared bivalve families with different symbiont transmission modes, citing a meta-analysis and some parts of the current study. Then, how much has been known about the transmission in the selected groups of bivalves? Differences in transmission modes ... is this an entirely new finding, or has there been prior data? After completing this review, I am still not sure.

With the above and below listed changes, and the additions made in response to Reviewer 1 and 2, we think we have clarified the issues presented here. Specifically, more information is provided about the associations, the use of the association for the question at hand (marine endosymbiont genome evolution) is better justified, and we have added a methodological flow chart (new S1 Fig.) to better acquaint the reader with how the data was obtained and analyzed.

I realize that the journal requires that the methodological details are only provided in the last section or/and in the supplement. Despite this, I would expect to find a summary of what the authors did at the end of the Introduction, or perhaps at the beginnings of paragraphs corresponding to Results. That was not the case here. It took me a substantial amount of effort (jumping between sections of the main text and the supplement) to understand that the authors have sequenced and analyzed gill metagenomes from multiple individuals from a total of eight populations of seven bivalve species. I further understood that for one individual per species, a

reference symbiont + mitochondrial genome was assembled, facilitating the use of lower-coverage data for the symbiont diversity comparisons within and across hosts. Despite my background in invertebrate symbiont diversity and genomics, I failed to understand some of the analyses. A summary of the experimental setup would have greatly helped me follow the text. Please make it easy for readers to understand what you did!

Thank you for letting us know that our methods were not conveyed clearly enough. As it would take quite a bit of space to provide a written overview of the methods outside of the Materials and Methods section (and still might confuse some readers), we decided to instead add a graphic overview of the methods in a new Fig. S1. This figure is referenced in the following new sentence at the end of the Introduction:

> "...Here, we use this model study system and a powerful population genomic approach (described in S1 Fig and S1-S3 Supporting Text), to show that homologous recombination is ongoing even in the most strictly vertically transmitted associations and may enable the maintenance of large and intermediate genome sizes indefinitely."

I am puzzled by some of the findings of the study, and their interpretation. The authors claim in the Abstract that they compare symbiotic systems "that range from primarily vertical to strictly horizontal transmission". However, the host-symbiont phylogeny comparison (Fig. 1C) does not make it clear that the differences among host species are significant. At the same time, I am surprised that when sampling replicate individuals from a population, the authors discovered substantial mitogenomic diversity. That is not a pattern I have seen in insects; is it expected in bivalves? The authors seem to be using the smaller genome sizes of vesicomyid symbionts, lower recombination rates and other genomic features as a secondary confirmation of the deducted mode of transmission. Once again, was the mode of transmission known before the study? If not, then what is the likelihood that the observed patterns are due to biological differences among hosts, for example in the intra-population diversity?

Thank you for further emphasizing the need for more background information about these associations. Extensive molecular, cellular, and phylogenetic evidence exists for the transmission modes of all of these associations. Our presentations of evidence consistent with the known mode more serve as internal controls, which we are very happy to have in a genomics study, rather than a secondary confirmation of transmission mode, as you suggest. Basically, we know the broad-scale transmission modes (i.e., average modes over time) for these groups, however, we do not know how rare events may have shaped their genome evolution. That is the point of this work: to apply existing symbiont genome evolutionary theory to fine-scale population processes to understand why some symbiont genomes do not degrade, despite their broad-scale transmission mode predicting it should do so. We think that the added background in the Introduction should help with these issues.
Regarding the similarity among genealogies in Fig 1C, we have added the following details to the first paragraph in the Results and Discussion (lines 152-163) that better explains this:

> "Comparative analyses of host mitochondrial and endosymbiont genome genealogies show strong evidence for horizontal transmission in all six populations (Fig. 1C). Despite this apparent similarity in transmission mode, the vesicomyid endosymbiont genomes are approximately one half the size of the solemyid or mytilid endosymbiont genomes, which themselves are approximately consistent with their free-living ancestors. Nonetheless, at 1-1.2 Mb, the partially degraded vesicomyid endosymbiont genomes are still ten times larger than the smallest terrestrial endosymbionts [1]. The genealogical discordance shown in Figure 1C indicates that horizontal transmission has occurred in the histories of these populations, however, it does not

suggest how much because concordance is eroded even by exceedingly low rates of horizontal transmission [43], saturating the signal genealogies can provide. Thus, a finer scaled exploration of symbiont genetic diversity was necessary to characterize the population-level processes influencing genome erosion."

As for the population-level mitochondrial diversity, this was not surprising to us at all. First, we should point out (in case it was missed in the figure legend) that these trees are cladograms, and so the branch lengths are meaningless. We did this because it is easier to show topological concordance/discordance between trees if they have equal heights. As listed in S4 Table, values of pi ranged from 3.47e-4 to 4.19e-3 and numbers of segregating sites in each mitochondrial population ranged from 31-327. Looking quickly at the literature values for other molluscs, this seems to be inline with what has been reported for cephalopods (Winkelmann et al. 2013) and can even be higher, as in Chaetognaths (Marlétaz et al. 2017). Thus, we do not see any reason to worry about the mitochondrial diversity values we obtained, which did involve variant filtering steps to ensure only high-confidence variants were included (as described in the Materials and Methods section).

I have not been familiar with folded allele frequency spectrum analyses, and I struggled to comprehend the plots in Figs 2-3 and S2-S4. Perhaps the authors could explain and discuss the analyses and patterns in a way that makes it easier for biologists not familiar with that particular method to understand the expectations and interpret patterns from these plots?

Folded allele frequency spectra are simply histograms of the allele frequency distribution, conditioning on the minor allele instead of the derived allele. Using the minor allele necessitates folding the spectrum because the polarity of the substitution is not known. Without knowing the ancestral state, a low frequency minor allele could either be a derived allele rising in frequency or an ancestral allele declining in frequency. To briefly cover some of this information in the text, we have added the following statement to the Materials and Methods section (lines 460-463):

> " ...As very closely related sister taxa have not been sampled for most of these bacterial genomes, ancestral/derived alleles could not be identified and we could not plot unfolded allele frequency spectra. Instead, folded allele frequency spectra were calculated for minor alleles and plotted in R. "

In the main text, the authors talk about vesicomyid, mytilid/bathymodiolin, and solemyid symbionts. In the figures, they provide Latin names of host species. I found it somewhat difficult to match and navigate the sets of names, and ended up searching multiple times which species belongs to which group. Perhaps the authors could think of ways of editing figures in a way that would help readers make the connection?

This is indeed a difficult issue to resolve. We tried to use extremely consistent colors, which we tested and determined to be discernible to a color blind reader, in order to avoid confusion. We bolded the family names in Fig. 1. and we tried adding additional labels to Figures 2-4 to emphasize which of the three families each species belongs to, but the added text made the figures look too cluttered. Are you sure the consistent coloration does not make the groups obvious? We have read through all of the figure legends to ensure they emphasize the association color code.

Finally, the authors use the genomic data for phylogenetic reconstructions. The addition of functional / contents analysis would have made the current story much stronger.

Thank you for this suggestion, however, the coding contents of highly related genomes have already been discussed in depth elsewhere (*e.g.*, Newton et al. 2007; Kuwahara et al. 2007; Dmytrenko et al. 2014; Sayavedra et al. 2015; Ponnudurai et al. 2017) and are not exactly relevant to the point we are trying to make. We are more interested in understanding the broad scale evolutionary pressures that shape symbiont genome evolution, rather than the specific functional properties shared, acquired, or lost from each genome. Thus, adding this analysis would be beyond the scope of this paper. However, this would be a great secondary study that should be completed after we release the genomes!

To sum up, I feel that the dataset holds strong promise for a publication. I strongly recommend that the authors present these data in the context of filling gaps in the understanding of bivalve symbioses. The systematic testing of whether the symbiont genomic patterns align with expectations for a given transmission mode would be a useful addition, but should not be the main focus. The authors need to explain in a clear and accessible way what was the state of the field, and which of the findings are new. They also need to state clearly what they did and why, how the findings complement existing knowledge, and what the results mean.

Thanks for your comments. We hope we addressed your concerns. Please let us know if we misinterpreted anything or you think additional clarification would be helpful.

Specific comments:
Lines 210-211: The authors wrote: "These results validate long-standing but untested theory and demand a reinterpretation of the vast diversity of symbioses...". I am finding the claim that "the vast diversity of symbioses" should be reinterpreted based on data for three families that largely conform to the expectations quite arrogant.

Thank you for your opinion. We in no way meant this statement arrogantly; we only mean that given the similarities of these associations to other symbioses, and the standing mysteries regarding larger genome sizes in marine endosymbionts, this association is likely very informative about a whole wealth of associations that have yet to be studied. We mean this similarly to how people refer to model systems when they are studying processes such as genetics, cell biology, or developmental biology in single organisms, such as fruit flies or nematodes, but applying their findings to our understanding of the processes generally. Since we definitely do not want to upset any of our potential readers, we changed the language to hopefully soften and qualify this statement as follows (lines 330-333):

> "These results validate long-standing but untested theory and suggest that the diversity of symbioses found to exhibit intermediate rates of horizontal transmission and incomplete genome degradation may be undergoing similar population-level processes."

Lines 231-233: Name kit/enzyme manufacturers

We have added these details.

Lines 233-234: "to uniquely label both i5 and i7 indexes for each sample that was sequenced on a single lane of a Hiseq4000. We sequenced a total of four lanes of Hiseq4000"... Before I read into the supplement, I was

quite confused. I initially understood that six spp. were sequenced, each filling its own lane. Change the wording.

Thanks for pointing out the poor phrasing here. We have corrected the wording to read:

> “...Indexed samples were pooled and sequenced on single lanes of a Hiseq4000. We sequenced a total of four lanes of Hiseq4000 paired-end 150 bp sequencing across the entire study. ...”

Lines 297-298: The authors wrote "After producing endosymbiont genome assemblies and host mitochondrial genome assemblies for each host/endosymbiont population..." . This is misleading and confusing: the assemblies were provided for a single host individual per population.

Thank you for pointing out this mis-phrasing. We have corrected this section to say that we sequenced genomes for each species.

Fig. 1C. What genes are the trees based on? Please provide scale bars. What are the outgroups?

These whole genome trees are actually scaleless cladograms, which enable easier topological comparison between trees because the tips are aligned. This is described in the figure legend. We have also added that these trees are based on whole genome data and are midpoint rooted to the figure legend as follows:

> “C) Mitochondrial and symbiont whole genome genealogies are discordant for all groups, indicating that sufficient amounts of horizontal transmission occur in vertically transmitted vesicomyid populations to erode the association between these cytoplasmic genomes. Maximum likelihood cladograms are midpoint rooted, and nodes below 50% bootstrap support are collapsed. Species are color coded by their symbiont transmission mode as in A).”

Fig. 4E. The sample labels are provided, but the font size makes them virtually illegible.

We have replaced this panel with dated phylogenies for host and symbiont in a separate figure, Figure 5.

Table S1. Why are the genome coverage values for nanopore reads provided as "na"?

We did not initially list the values because these reads were used for symbiont genome scaffolding only, and not base-calling for which coverage is more relevant. However, for completeness, we have included the Nanopore coverage for symbionts in the S1 Table. As we did not map the long reads to the mitochondrial genomes or use them for assembly, we reported those coverages as “not mapped”.

Table S2. What is the advantage of providing the genome, scaffold etc. sizes in the format "2.37E+06" as opposed to the actual values?

This was just to save space when formatting the tables to fit in the text. We have reformatted the numbers to not be in scientific notation.

In Table S3, the authors provide the list of "Symbiont species named in this study and named previously", including newly proposed names. The table is only referenced once in the main manuscript, but no information about that nomenclatural aspect is provided.

Thank you for pointing this out. We have added a section to the Materials and Methods and S3 Supporting Text where the genera are diagnosed and the species are described.

Also, I am not a Latin expert, but I believe that the proposed symbiont names are incorrect grammatically. The generic names should be nouns in the nominative, singular form, with endings corresponding to declension - but as far as I can tell, several of the generic names proposed here do not conform to these conventions. Please consult the bacterial nomenclature rules.

Thank you for bringing this to our attention. While tentative naming with the Candidatus designation is not subject to the Prokaryotic Code regulations, we would prefer if these names were technically valid. In the event the symbionts are able to be properly cultured and deposited as type strains someday, adhering to the nomenclature rules would enable these names to be validated, sans the Candidatus designation. We have corrected the names to comply with the regulations that must be satisfied for name validation (S3 Table and S3 Supporting Text).
