# Supplementary material for: Horizontal transmission and recombination maintain forever young bacterial symbiont genomes
Source: PLoS Genet. 2020 Aug 25;16(8):e1008935. doi: 10.1371/journal.pgen.1008935 (PMC7473567; doi:10.1371/journal.pgen.1008935)
Supplement: Attachment — Submitted filename: r2r-2-ForeverYoung_PLoSGenetics.pdf [file pgen.1008935.s024.pdf]

Re (MS): PGENETICS-D-19-01871R1 (revised manuscript)
Russell, Pepper, Svedberg, Byrne, Ruelas Castillo, Vollmers, Beinart, and Corbett-Detig
Horizontal transmission and recombination maintain forever young bacterial symbiont genomes

Dear PLoS Genetics Editors,

Thank you for this second round of helpful comments and reviews on our manuscript. We are happy to hear that we resolved many of the reviewers' concerns in the first round of revision, and hope we have adequately addressed the remaining concerns in this iteration. Our responses to each of the reviewers' comments (in italics) are listed below.

Additionally, we would like to thank newly added Reviewer 4 for their many astute and interesting suggestions for additional analyses. However, many of these were beyond the scope of this paper, which specifically aims to understand the population genetic processes underlying genome erosion in symbionts with different transmission modes. Analyses to ascertain the precise functional consequences, inducing potential constraints and feedbacks, underlying genome erosion would require functional data and at least as much space as we currently have dedicated to our results. So, we will have to pursue this work in a subsequent publication.

We have also submitted the genome assemblies (symbiont and mitochondrial) and the raw sequencing data to NCBI, under the accessions listed in S1 Table. These data will be publicly available as soon as NCBI finishes processing them (some are already available).

Thank you again for taking the time to help improve our manuscript. We greatly appreciate your and the reviewers' efforts.

Shelbi Russell

Shelbi Russell, Ph.D.
University of California Santa Cruz
shelbilrussell@gmail.com

Notes from the Editors:
The reviewers appreciated the attention to an important topic but identified some aspects of the manuscript that should be improved.

We therefore ask you to modify the manuscript according to the review recommendations before we can consider your manuscript for acceptance. Your revisions should address the specific points made by each reviewer.

Reviewer's Responses to Questions
Comments to the Authors:

Please note here if the review is uploaded as an attachment.

Reviewer #1: I thank Russell et al for their thoughtful and extensive response to my previous comments. The phylodynamic analyses in Figure 5 are excellent, and a valuable addition – to me, it would seem helpful to have this as Figure 1, as it allows a non-specialist reader to immediately understand how divergent the host and endosymbionts are, and the durations of their associations. The validation of the recombination analysis method is also nicely done, although there are a couple of aspects it would be useful to address in point 2 below.

Thank you for this suggestion. We agree that moving former Figure 5 to an earlier point in the text helps to communicate the important concepts in the paper. We have moved the phylogenomic dating figure (former Figure 5) up to be the second figure, and we have adjusted the Results and Discussion text accordingly. We tried making it Figure 1, but found that we needed to introduce that figure first to communicate information about the groups' transmission modes and genome sizes.

The remaining problem is not concerning the data, but rather the interpretation of the link between recombination, selection and genome structure. Given the authors' answers to my first review, there are three questions that it seems important to address:

*(1) As the authors state all recombination they refer to is homologous recombination, is the rate high relative to other species? The authors state, "these populations have some of the largest effective recombination rates ever reported for bacteria" – this is unaltered from their first draft. Yet the authors state in their response that they "conflated" rho*l/theta with r/m (with the former giving higher estimates of the impact of recombination) – yet the cited Vos & Didelot study reports r/m, making a comparison of these different statistics very difficult. Given their novel method predicts recombination makes a bigger impact than mutation in the Calyptogena magnifica symbiont, despite it lacking the requisite homologous recombination machinery, can the authors validate this claim, and provide a robust comparison with other bacterial species, using existing software (e.g. ClonalFrameML, chromosome painting or Gubbins)?*

> Thank you for pointing out that we need to more clearly explain the validity of our recombination rate comparisons. Fortunately, Ansari and Didelot 2014 worked out the mathematical relationship between r/m and rho*I. We added the following text to the manuscript to clarify how our parameters compare to r/m, and what that means for relative recombination rates (lines 242-248):
>
> > "Given that these recombination events are occurring within symbiont populations ($\theta_{recombinant} = \theta_{genome}$), our estimate of rho*I is equivalent to r/m from previous studies of bacterial

recombination rates (r/m = rho*l*$\theta_{recombinant}$ /θ_{genome}; see [51]). Comparing to r/m values across bacteria and archaea, which range from 0.02 to 63.6 [52], reveals that these symbiont populations have some of the largest effective recombination rates ever reported for bacteria (rho*l from the B. septemdierum symbionts equals 46.3, which is in the 96th percentile of previously measured rates)."

Unfortunately, we cannot use programs such as ClonalFrameML or Gubbins on these data, as they perform poorly on populations with high recombination rates and low sequence diversities. This is for a couple of reasons: first, these programs usually assume that recombinant regions have higher sequence divergence than the clonal frame, which is not true for our data because recombinants come from within the population. Second, these programs cannot account for overlapping recombination events, which are unavoidable at high recombination rates in these small genomes. These dense overlapping events break up and obscure the clonal frame. Supporting the fragmented clonal frame in our data, Russell et al. 2017 ISME J shows that the *S. velum* symbiont displays very different genealogies across sliding windows of its genome (figure S7). Taken together, these issues would likely cause these methods to underestimate recombination rates. These are important caveats and are reported in figure 2 of the ClonalFrameML paper (Didelot and Wilson 2015). Chromosome painting also will not work on our data, as that method models meiotic recombination (crossing over), and not bacterial recombination (gene conversion). These issues with the previously developed methods for detecting recombination were why we developed our own method and validated it using the *B. cereus* dataset, which has been run on the widest range of methods of any dataset.

Regarding how recombination can occur in a genome lacking traditional recombination genes such as recA, other recombination mechanisms exist, which the symbionts may be using. We explain this in the following sentence added to the text (lines 248-252):

> "Despite their lack of many genes normally required for recombination [24], we found evidence for modest rates of recombination within both partially degenerate vesicomyid genomes. This capability may be enabled via "illegitimate" mechanisms, e.g., RecA-independent recombination via slipped-mispairing or single strand annealing [24,53]. "

I am also confused about the statement in the response, "When a gene conversion event occurs on a genealogy, it creates an additional lineage within the converted segment to the right and to the left of the recombination breakpoints." Why is an additional lineage created either side of a gene conversion event – both the upstream and downstream regions are part of the same clonal frame?

It is not necessarily true that either side of a homologous recombination/gene conversion event are part of the same clonal frame. For example, this assumption would be false under high recombination models with overlapping gene conversion events that iteratively replace ancestral and introduced material. *E.g.*, when recombination is widespread. The ability of FastSimBac to model these types of events is one of the reasons we choose it as our coalescent simulator. To clarify that we are merely describing how recombination is modelled in FastSimBac, we've added the following (in bold) to S2 Supporting Text:

> "Throughout, we model recombination as equivalent to **occasionally overlapping** gene conversion **events** in eukaryotic species. **Moving along a chromosome from left to right**, when a gene conversion event occurs on a genealogy, it creates an additional lineage within the converted segment to the right and to the left of the recombination breakpoints **(as described in [111])**. **If no additional gene conversion events occur within the gene conversion tract,**

then the sampled genome returns to the previous marginal genealogy when the tract ends.“

(2) Do the authors think recombination prevents, or causes, genome degradation? The Results section ends, “Thus, ample time has passed for these symbiont genomes to erode, but horizontal transmission and recombination have likely prevented it.” – an inference emphasised in the Conclusion – which suggests to the reader that recombination directly limits genome degradation. Yet in the Results section, the authors suggest a “recombinational processes may partially underlie genome erosion”, as “a rare recombination event induces a deletion that drifts to high frequency” – which is the process of genome degradation. As the authors say in their response, “Whenever we refer to recombination, we mean homologous recombination”, this suggests faster homologous recombination (as seen in some of these endosymbionts) should drive faster genome degradation. Can deletions not be spontaneous, rather than occurring through recombination? Might selection not be more important than recombination in the observed differences?

Thank you for pointing out that we need to clarify the points we are trying to make, as this isn’t an either/or model. We think that recombination is involved in both causing and preventing genome degradation. The mechanism of recombination can produce genomic deletions that may drive genome degradation. Of course, other mechanisms can cause deletions too (*e.g.*, replication slippage). If recombination (or any deletion-generating mechanism) is paired with gene flow from horizontal transmission, then a deleterious deletion can be rescued by recombining with a functional copy of the deleted region. A deletion could be retained if it is selectively advantageous, however, the vast majority of deletions are probably deleterious due to loss of functionality. So, while recombination could increase rates of genome reduction over short-term time scales, once the genes required for recombination are lost, then recombination-induced deletions/rearrangements will stop too. That is the point where we think the vesicomyid symbiont genomes are now.

Thus, the rescue function of recombination is dependent on the symbiont effective population size being large. Selection can only act if there is genetic variation to act upon. Horizontal transmission serves to introduce rescuing genetic variation, which can be integrated into the resident genome through homologous recombination to increase the variation present in the symbiont population. Viewing selection as distinct from recombination in the adaptive process is incorrect, as recombination serves to facilitate selective efficacy and precision (e.g., with high enough recombination rates, a single beneficial mutation can be selected, independent of the genomic background it evolved on). This was the purpose of Figure 4 (former Figure 3) and the following sentence (lines 258-260): “A fundamental consequence of decreased homologous recombination rates for endosymbionts is that selected mutations cannot be shuffled to form higher fitness chromosomes and remain linked to neutral mutations for longer times.”

We have tried to clarify these concepts further by adding a new explanatory figure, Figure 6, and the following details to the text (additions to existing text in bold).

Lines 285-288: “Given the role of recombination in altering bacterial genome structure ([56]; illustrated in Fig. 5A), and the signals of **recombination and** linked selected sites in the vesicomyid symbionts (Fig**s. 3D and** 4F-G, **respectively**), recombinational processes may partially underlie genome erosion**, in addition to preventing it**.”

Lines 289-291: "This could proceed in the following way: first, a rare recombination event induces a deletion that drifts to high frequency in the within-host population **(other events can also induce deletions, such as strand slippage [57])**. "

Lines 326-328: "Thus, ample time has passed for these symbiont genomes to erode, but horizontal transmission and recombination have likely prevented it **(as depicted in Figure 6)**"

Figure 6 legend: "Model of endosymbiont genome degradation prevention via horizontal transmission and recombination. Horizontal transmission of symbiont genotypes between hosts and recombination between genotypes in mixed infections are able to introduce sufficient levels of genetic diversity to prevent or delay genome degradation by restoring functional versions of mutated or deleted regions. Prevention can continue until recombination capabilities (RecA-dependent and independent) are completely lost, at which point, genetic rescue is no longer possible without wholesale symbiont replacement."

(3) To what extent is the study describing the evolution of symbionts? The addition of the tree is excellent, and highlights those comparisons between endosymbionts that are sister taxa (and can reasonably be assumed to have maintained an endosymbiotic lifestyle over the period of their divergence), and those that are distantly related, and therefore are likely to have evolved as free-living bacteria over some proportion of their divergence. The authors accurately refer to "Highly divergent solemyid symbionts" in the Results, but it would be more helpful to the reader to point out that it cannot be assumed the divergence occurred as symbionts (unless the many related taxa have all independently evolved to be free living), particularly given the pairwise comparison in Figure 4C is contrasted with that in 4D, where the compared isolates are a single clade, the most recent common ancestor of which was likely an endosymbiont.

We completely agree that we do not know the ancestral state for the solemyid symbionts. However, we do describe the potential for the solemyids to have evolved from free living ancestors earlier in the manuscript on lines 189-192: "While host-switching and novel (free-living) symbiont acquisition have certainly occurred in Solemyidae, and our data indicates such an event may have happened after *S. velum* and *S. pervernicosa* diverged (Fig. 2A), it may be relatively rare across geological time."

To help clarify, we've added the bolded phrase to the following sentence:

"While host-switching and novel **(free-living)** symbiont acquisition have certainly occurred...."

I reiterate that I think this is an interesting and valuable study, but given its complexity, it is important to clarify and assess the central message.

Minor points:
In some places in the updated text, "mya" is just "my".

These have been corrected.

Reviewer #2: The authors improved the clarity of the manuscript and added a nice analysis of the estimated divergence dates for both the hosts and symbionts. They also answered and implemented all of my questions/suggestions which I appreciated. However, I still believe the 'forever young' term in the title is stretching it a bit since this term only refers to the symbionts up to this point in time. It's difficult to predict what will happen to the symbionts in the future, especially if recombination rates and horizontal transmission

decrease. Therefore, they may not be 'forever young'. I still think this paper is invaluable to the symbiosis field, and should be accepted since very few studies on symbiont population genetics exist from hosts other than insects.

> Thank you for your comments and your one last criticism. While we see your point about the title, we like its whimsy and do not think readers will be misled into thinking we have somehow figured out how to predict the future evolutionary trajectory of symbiont genome evolution (we wish we could do that!). Furthermore, any declarative title could come off as "predictive". For example, the alternate title "Horizontal transmission and recombination prevent bacterial symbiont genome degradation" could allude to indefinite prevention. So, if possible, we would like to keep our original title, but we would be willing to reconsider if the editor feels strongly that we should change it.

Reviewer #4: This study by Russell et al. uses a population and comparative genomics approach to examine differences in the endosymbiont population dynamics among 3 families of marine bivalves with different modes of symbiont transmission. Each symbiosis is independently evolved with endosymbionts providing chemosynthetic carbon fixation. Overall I like the study and I think it represents a significant contribution to the field of marine bacterial endosymbioses. The data generated represents a valuable resource to the broader scientific community. Additionally, after reading previous reviews I feel like the authors have addressed the most significant issues in the manuscript. I have comments.

It isn't clear to me (and this could be because I am less familiar with marine endosymbionts) why there seems to be an expectation that these symbiont genomes should undergo the same degree of erosion as insect endosymbionts?

> As we explain in the introduction, genome erosion is the predicted outcome of long-term obligate bacterial symbioses in general. This theory was developed using aphid symbionts as a model, but it is not supposed to be specific to those bacteria, as it is based on population genetic principles. The lack of extreme degradation in many of the vertically transmitted marine associations prompted us to ask why, which is the purpose of the paper. With our addition, the theory of endosymbiont genome evolution includes provisions for population parameters more commonly encountered in marine associations than terrestrial. We have tried to clarify that this is a general theory (instead of one specific to insects) with the following addition (in bold):
>
> > "While the degraded genomes of many endosymbionts are consistent with this **general** theory, such as symbionts of terrestrial sap and xylem-feeding insects [1], a diversity of associations present discrepancies."

1) The host is gutless, and relies entirely on the symbiont for ALL of its nutritional needs, not just a handful of essential amino acids. This necessarily implies the lower bound for genome size will be much higher than for an obligate terrestrial endosymbiont, where in drift will rule the drift/selection balance for a much higher fraction of the genome.

> It is true that the host relies on symbiont metabolism for all of its nutritional needs, however, this does not necessitate an enormous number of genes. These symbionts are like chloroplasts in many ways - they fix inorganic carbon and synthesize amino acids. While chloroplast genomes are larger than mitochondrial (~200 Kb vs ~20 Kb), they are still much smaller than those of free-living bacteria. A formal comparative analysis of the minimal gene content required to perform various symbiotic functions would be interesting, but is beyond the scope of this paper.

2) Line numbers.

3) Drift is much stronger in terrestrial endosymbionts that are never exposed to an outside environment. It seems marine symbionts, even in the case of mostly vertical transmission, are still more exposed to the environment as they are essentially processing the immediate environment for the host. Additionally, given rates of horizontal transfer, they clearly have to survive in some form outside the host. Whether, the transfer events are mediated through, burst, unfertilized eggs, the contents of which are then filtered by the gills of a neighbor or otherwise, this is far beyond the level of exposure transovarially, internally fertilized terrestrial arthropod symbionts will experience. I feel the more apt comparison is among ectosymbionts of terrestrial arthropods.

In theory, marine intracellular vertically transmitted symbionts are never exposed to an outside environment, but as shown in Russell 2019, the marine environment may indeed predispose symbioses to higher rates of horizontal transmission than terrestrial environments do. That is what we mean when we say (lines 103-105): “Symbioses in marine environments exhibit significantly more horizontal transmission between hosts than those in terrestrial environments [25], suggesting that symbiont gene flow between hosts may prevent genome decay...”

We are not sure what you meant by the statement “symbionts … are still more exposed to the environment as they are essentially processing the immediate environment for the host”. These are intracellular symbionts, so at the cellular/organismal level, they should be as isolated from the environment as the intracellular insect gut symbionts. Even comparing gills to guts is similar: both organs are in close contact with flow from the external environment.

In regards to whether this is the proper system to be investigating genome evolution in: this paper is not attempting to be a comparison to insect symbionts, but instead, an exploration of what may be influencing genome size in marine endosymbionts. Since the theory of symbiont genome evolution is based on population genetic principles, it is used to explain processes occurring in all associations, despite being originally developed for the insect endosymbionts. So, our comparisons to those systems are only in regard to us porting the theoretical predictions over to more permissive horizontal transmission regimes. We have tried to describe this in the text (as detailed for your first comment above), but would welcome suggestions on how to make this point clearer.

4) Line numbers.

5) Despite these points, as the previous reviewer pointed out, Mueller’s ratchet will ratchet more in the vertically transmitted symbionts, and the data support this (dN/dS, minor allele frequency spectrum). However, the maintenance of diverse coding content likely wouldn’t persist for 10s or 100s of millions of years if it weren’t needed, thus I see degradation to an organellar-like state as somewhat less likely than what occurs in other systems. Perhaps the most surprising aspect is that host-symbiont cospeciation and associations persist for as long as they do without replacements.

We have tried to show here that if horizontal transmission and recombination rates are high enough, these processes may be sufficient to maintain diverse coding content over long periods of time. It was never our intent to ascertain what genes are “necessary” or to show how the loss of particular genes feeds back on processes that can maintain those genes. To do so would likely require more functional data than we have or can generate for these difficult to sample species (most of which occur in the deep sea). Perhaps in the future, we could identify the genes required for horizontal transmission and

non-recA-mediated recombination, and then evaluate whether the loss of those precipitates accelerated genome decay. However, at this point in time, those questions are not addressable for this system.

This comment also appears similar in concept to your previous one regarding the assumption that these chemosynthetic symbionts need more genes than other symbionts do. As discussed for comment 1 above, we do not think that is necessarily a correct assumption, but would need to be tested explicitly, which warrants a separate study. Moreover, our study emphasizes a comparison between highly recombining and lowly recombining endosymbiont populations. The differences in outcome for genome diversity are therefore likely driven in large part by the population genetic processes we study here.

6) Line numbers.

7) It could likely be argued that the host takes over much of the workload as the symbiont degenerates, as seen in other systems. Delegation of these functions presumably stops at carbon fixation +other complex nutritional provisioning steps (like amino acid synthesis). The point of the prior reviewer about the remaining coding capacity is relevant in this context.

It is indeed interesting to consider symbiont functions that may have been relegated to the host. A couple of these (or closely related) symbiont genomes and inventories of their genes have been previously published (Newton et al. 2007; Kuwahara et al. 2007; Dmytrenko et al 2014; Sayavedra et al. 2015; Ponnudurai et al. 2017), so we would rather not repeat those analyses here. Furthermore, this question is beyond the scope of the paper, as we are not investigating how symbiont functions are accomplished during genome erosion. Instead, the goal of this paper is to understand the population genomic processes underlying symbiont genome erosion.

Other comments.

Figure 1C- Fst as well as other data being what it is among the symbionts associated with these bivalves, I have no doubt there is gene flow between hosts as this figure is meant to convey. However, with rampant polytomies in the trees, the degree of transfer might not be nearly as prevalent as this figure suggests (I see several ways of making it more concordant at a glance). I understand that you point this out in the text, and that this figure is more or less supposed to be a toy model to make a point about concordance, however, this figure doesn't support the point that gene flow erodes host/symbiont concordance because it tells us little about the relationships that are supposedly "discordant" due to gene flow. Also, gene flow and recombination erode our ability to produce fully resolved phylogenies. These are trees made from populations, with a single consensus likely representing the major allele. With lots of gene flow and recombination it isn't likely one would resolve these in any meaningful way at the population level but a polymorphism aware model (such as the one implemented in IQ-Tree2) run on allele frequency data would be a big improvement. The model allows for fixation of heterozygosity with Watterson's theta or specifying the distribution from which to draw states. As for the mitogenome trees, I would use the ML tree without collapsing branches (maybe partition by codon position in the future), low support is expected with so few segregating sites and a single locus, but knowing the "best" hypothesis is still more informative than polytomies.

You are correct in your evaluation of Figure panel 1C as more of a toy model of the genealogical effects of horizontal transmission than a tool to precisely view phylogenetic discordance. As we describe in the

paper (lines 155-159), topological concordance is eroded by very low rates of horizontal transmission. So, this was merely a preliminary test to graphically show that horizontal transmission does occur within populations, and is not a rare event for vesicomyids (previous studies (*e.g.*, Stewart et al. 2008) found discordant gene trees between vesicomyid symbiont species using just a handful of markers). We are not implying anything about overall rates of horizontal transmission with this figure, due to the saturation of discordance (see Brandvain et al. 2011 for the underlying theory for this). We re-examined our mapping of the co-phylogenies and identified a few nodes that could be rotated for clearer viewing of the relationships. We have made these changes, although, we would like to emphasize that this makes no difference in our interpretations, as these taxa are strictly horizontally transmitted.

We appreciate your efforts to recommend strategies for us to improve the genealogies presented in Figure 1C. However, there are a couple of reasons that re-running the phylogenetic inference will be unlikely to be successful. First, the mitochondrial genomes are clonal, so recombination cannot explain the polytomies in the mitochondrial genealogies. Instead, the lack of resolution is due to low sequence diversity (pairwise diversity 3e-4 to 4.19e-3), which we cannot remedy, as it is a characteristic of these population data. Thus, it is unlikely that another maximum likelihood inference method with more parameterization would produce a significantly improved tree with these limited data.

Regarding the symbiont genome, we do know from our earlier work (Russell et al. 2017), that genealogies inferred from sliding windows across the genome do have very different topologies. Many of these trees were well-resolved, suggesting that the polytomies are due to conflicting evolutionary histories of different genomic segments, and not some flaw in tree inference. Repeating this and conducting a finescale analysis of recombination rate by region is a great idea, but is beyond the scope of this work.

Lastly, we respectfully disagree with your opinion that it is better practice to present fully resolved phylogenies, even if they are not well-supported. We collapsed nodes with bootstrap values below 50%. Bootstraps below that value indicate that the node was not recovered a majority of the time, so presenting can be misleading. Perhaps this is a matter of philosophy, but we would prefer to only present the topologies that the data confidently support. Furthermore, the trees have adequate numbers of resolved nodes to illustrate our point: that mitochondrial and symbiont phylogenies are not concordant as would be expected if these two cytoplasmic components were always transmitted together through the germline. Resolving a few more nodes would not change that result given the resolved discordance at deep nodes.

Figure 2C- The solemyids seem odd. I understand by looking at the supplementary that this is meant to represent the fact that they are intermediate. However, some individuals of S. pervernicosa almost appear to be in balancing selection? This appears to hold for the minor allele freq spectrum of individuals as well as Tajima's D. I guess this would be the case of drift or an individual that had gene flow restricted for a number of generations and then recently had a transfer event. I guess my question is whether you see in nonsense mutations on one haplotype background that is compensated for in the alternative haplotype and vice versa? Since you have nanopore and read backed phasing data, this might be interesting to look at.

Thank you for this suggestion. Indeed, the within-host dynamics of the solemyid symbionts are fascinating. S. Russell has actually previously published a paper that investigates the within-host *S. velum* symbiont genetic diversity using short-read data (Russell and Cavanaugh 2017). From this work, it does appear that the allele frequency spectra have been shaped by horizontal transmission with

recombination and inheritance. It is a great idea to use the long-read data to explore this idea in the other symbionts. However, to keep the point of this paper focused on the drivers and constraints of symbiont genome erosion, we will have to save these analyses for another study.

I have attached a version with line numbers that has additional comments.

Thank you for catching these typos. I have a comment about one of your comments:

Comment on insect genome sizes (line 60): I cannot find mention of a ~1 Mb genome size in Bennett and Moran 2013 GBE, so the citation was left as-is.
